# Chronic Airflow Limitation, Emphysema, and Impaired Diffusing Capacity in Relation to Smoking Habits in a Swedish Middle-aged Population

**DOI:** 10.1513/AnnalsATS.202402-122OC

**Published:** 2024-12-01

**Authors:** Anders Blomberg, Kjell Torén, Per Liv, Gabriel Granåsen, Anders Andersson, Annelie Behndig, Göran Bergström, John Brandberg, Kenneth Caidahl, Kerstin Cederlund, Arne Egesten, Magnus Ekström, Maria J. Eriksson, Emil Hagström, Christer Janson, Tomas Jernberg, David Kylhammar, Lars Lind, Anne Lindberg, Eva Lindberg, Claes-Göran Löfdahl, Andrei Malinovschi, Maria Mannila, Lars T. Nilsson, Anna-Carin Olin, Anders Persson, Hans Lennart Persson, Annika Rosengren, Johan Sundström, Eva Swahn, Stefan Söderberg, Jenny Vikgren, Per Wollmer, Carl Johan Östgren, Jan Engvall, C. Magnus Sköld

**Affiliations:** ^1^ Department of Public Health and Clinical Medicine, Umeå University, Umeå, Sweden;; ^2^ Section of Occupational and Environmental Medicine, School of Public Health and Community Medicine,; ^3^ COPD Center, Department of Internal Medicine and Clinical Nutrition,; ^4^ Department of Molecular and Clinical Medicine, Institute of Medicine, and; ^5^ Department of Radiology, Institute of Clinical Sciences, Sahlgrenska Academy, University of Gothenburg, Gothenburg, Sweden;; ^6^ Department of Occupational and Environmental Medicine,; ^7^ COPD Center, Department of Respiratory Medicine and Allergology,; ^8^ Clinical Physiology,; ^9^ Department of Radiology, Region Västra Götaland, and; ^10^ Department of Medicine, Geriatrics and Emergency Medicine, Östra Hospital, Sahlgrenska University Hospital, Gothenburg, Sweden;; ^11^ Department of Clinical Physiology,; ^12^ Department of Cardiology, and Clinical Genetics, and; ^13^ Department of Respiratory Medicine and Allergy, Karolinska University Hospital, Stockholm, Sweden;; ^14^ Department of Clinical Physiology,; ^15^ Department of Clinical Science, Intervention, and Technology,; ^16^ Department of Molecular Medicine and Surgery,; ^17^ Department of Clinical Sciences, Danderyd University Hospital,; ^18^ Department of Clinical Sciences, Huddinge University Hospital, and; ^19^ Respiratory Medicine Unit, Department of Medicine Solna and Center for Molecular Medicine, Karolinska Institute, Stockholm, Sweden;; ^20^ Department of Clinical Sciences Lund, Respiratory Medicine, Allergology, and Palliative Medicine, Faculty of Medicine, and; ^21^ Department of Translational Medicine, Lund University, Lund, Sweden;; ^22^ Cardiology,; ^23^ Respiratory, Allergy, and Sleep Research,; ^24^ Clinical Epidemiology,; ^25^ Clinical Physiology,; ^26^ Department of Medical Sciences, and; ^27^ Uppsala Clinical Research Center, Uppsala University, Uppsala, Sweden;; ^28^ Department of Health, Medicine, and Caring Sciences,; ^29^ Department of Clinical Physiology,; ^30^ Wallenberg Centre for Molecular Medicine,; ^31^ Centre of Medical Image Science and Visualization,; ^32^ Department of Radiology,; ^33^ Department of Respiratory Medicine in Linköping, and; ^34^ Department of Cardiology, Linköping University, Linköping, Sweden; and; ^35^ The George Institute for Global Health, University of New South Wales, Sydney, New South Wales, Australia

**Keywords:** chronic obstructive pulmonary disease, smoking, emphysema, impaired Dl_CO_, respiratory symptoms

## Abstract

**Rationale:**

Chronic obstructive pulmonary disease (COPD) includes respiratory symptoms and chronic airflow limitation (CAL). In some cases, emphysema and impaired diffusing capacity of the lung for carbon monoxide (Dl_CO_) are present, but characteristics and symptoms vary with smoking exposure.

**Objective:**

To study the prevalence of CAL, emphysema, and impaired Dl_CO_ in relation to smoking and respiratory symptoms in a middle-aged population.

**Methods:**

We investigated 28,746 randomly invited individuals (52% women) aged 50–64 years across six Swedish sites. We performed spirometry, Dl_CO_ testing, and high-resolution computed tomography and asked for smoking habits and respiratory symptoms. CAL was defined as post-bronchodilator forced expiratory volume in 1 second divided by forced vital capacity (FEV_1_/FVC) < 0.7.

**Results:**

The overall prevalence was 8.8% for CAL, 5.7% for impaired Dl_CO_ (Dl_CO_ < LLN), and 8.8% for emphysema, with a higher prevalence in current smokers than in ex-smokers and never-smokers. The proportion of never-smokers among those with CAL, emphysema, and impaired Dl_CO_ was 32%, 19%, and 31%, respectively. Regardless of smoking habits, the prevalence of respiratory symptoms was higher among people with CAL and impaired Dl_CO_ than those with normal lung function. Asthma prevalence in never-smokers with CAL was 14%. In this group, asthma was associated with lower FEV_1_ and more respiratory symptoms.

**Conclusions:**

In this large population-based study of middle-aged people, CAL and impaired Dl_CO_ were associated with common respiratory symptoms. Self-reported asthma was not associated with CAL in never-smokers. Our findings suggest that CAL in never-smokers signifies a separate clinical phenotype that may be monitored and, possibly, treated differently from smoking-related COPD.

Chronic airflow limitation (CAL) is defined as impaired expiratory airflow that does not normalize after bronchodilation or any other therapy, whereas the diagnosis of chronic obstructive pulmonary disease (COPD) requires symptoms in addition to CAL (1). Data on CAL prevalence in the general population are limited. Studies have indicated a great underdiagnosis and that post-bronchodilation spirometry is often not performed (2). Recent data from the SCAPIS (Swedish Cardiopulmonary Bioimage Study) pilot study (3), including 1,050 individuals aged 50–64 years, indicated a prevalence of CAL of 10.0% and 9.5% according to the Global Initiative for Obstructive Lung Disease (GOLD) and lower limit of normal (LLN) based on Swedish reference values (4), respectively. Based on the GOLD criterion (1), the international BOLD (Burden of Obstructive Lung Disease) study presented an overall prevalence of post-bronchodilation CAL of 10.1% (11.8% for men and 8.5% for women [5]), and similar data were reported from the Latin American PLATINO (Chronic Obstructive Pulmonary Disease in Five Latin American Cities) Study (6).

Although computed tomography (CT) has been used in studies including smokers and patients with COPD (7, 8), data on emphysema prevalence in population-based studies are limited. Also, population-based data on diffusing capacity, commonly reduced in emphysema, are rare. In the Canadian CanCOLD (Canadian Cohort Obstrucitve Lung Disease) study, diffusing capacity was significantly reduced in ever-smokers with CAL, whereas no reduction was seen in never-smokers with CAL (9).

Although cigarette smoking is the most common cause of CAL, the proportion of never-smokers in populations with CAL ranges between 20% and 50% (10–12). Known risk factors for CAL, besides smoking, are asthma, exposure to air pollution, and early events such as prematurity or viral infections in childhood (12, 13). These individuals have a substantial risk of morbidity in terms of hospitalizations, although low levels of inflammatory biomarkers have been reported (2). As the prevalence of smoking decreases in the western world, including Sweden, the proportion of never-smokers among individuals with CAL is likely to increase.

The mechanisms underlying chronic airway limitation in never-smokers are currently unknown, and data on clinical characteristics, demographics, symptoms, and imaging are scarce (12, 13), thus more detailed clinical phenotyping of CAL in never-smokers is warranted. The aim of the present investigation was therefore to study the prevalence of CAL, emphysema, and impaired diffusing capacity together with clinical characteristics in relation to smoking habits in a large middle-aged general population-based sample.

## Methods

### Study Sample

SCAPIS is a population-based prospective cross-sectional study (www.scapis.org). Between 2013 and 2018, 30,154 men and women aged 50–64 years were randomly recruited from the Swedish general population at six sites (Gothenburg, Linköping, Malmö/Lund, Stockholm, Umeå, and Uppsala), using the census register (14). The study was ethically approved as a multicenter study (# 2010-228-31M), and the participants gave their written informed consent.

**Figure 1.**
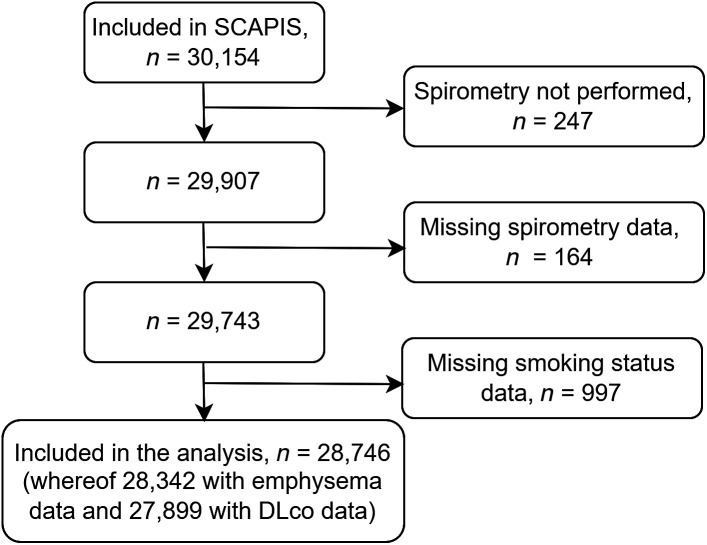
Flowchart of inclusions and exclusions of study participants. Dl_CO_ = diffusing capacity of the lung for carbon monoxide; SCAPIS = Swedish Cardiopulmonary Bioimage Study.

Study inclusions and exclusions for the present study are detailed in Figure 1 and characteristics of study participants in Table 1. All participants completed an extensive questionnaire on tobacco use, symptoms, comorbidities, and educational degree.

**Table 1.** Characteristics of the study population

Characteristic	All (*N* = 28,746)	Women (*n* = 14,813)	Men (*n* = 13,933)
Age, yr	57 (54–61)	57 (54–61)	57 (54–61)
Smoking status			
Current smoker	3,688 (13)	1,904 (13)	1,784 (13)
Ex-smoker	10,500 (37)	5,786 (39)	4,714 (34)
Never-smoker	14,558 (51)	7,123 (48)	7,435 (53)
Pack-years of cigarette smoking	12 (5.3–23)	12 (5.0–22)	13 (5.5–25)
Body mass index, kg/m^2^	26 (24–29)	26 (23–29)	27 (25–30)
hsCRP > 3 mg/L	4,684 (16)	2,612 (18)	2,072 (15)
Hemoglobin, g/L	141 (133–150)	134 (129–140)	149 (142–155)
Diabetes mellitus	2,107 (7.3)	804 (5.4)	1,303 (9.4)
Ischemic heart disease	706 (2.5)	194 (1.3)	512 (3.7)
CACS > 100	5,438 (20)	1,593 (11)	3,845 (29)
Asthma	1,461 (5.2)	834 (5.8)	627 (4.6)
Educational degree			
Comprehensive school	2,667 (9.3)	1,209 (8.2)	1,458 (11)
Upper secondary education	13,023 (45)	6,283 (43)	6,740 (49)
University	12,944 (45)	7,269 (49)	5,675 (41)

*Definition of abbreviations*: CACS = coronary artery calcifications score; hsCRP = high-sensitivity C-reactive protein.

Data are given as median (interquartile range) or *n* (%).

Height and weight were measured at enrollment, and body mass index was calculated as measured weight/height^2^ (kg/m^2^) (Table 1). Asthma was defined as self-reported “physician-diagnosed asthma with onset before 40 years of age,” based on the Global Strategy for Asthma Management and Prevention (https://ginasthma.org) and Reference 15. Diagnosis of diabetes was derived from self-reported diabetes or a fasting plasma glucose ≥7.0 mmol/L or hemoglobin A1c ≥48 mmol/mol at baseline. Ischemic heart disease was defined as self-reported physician-diagnosed angina pectoris, myocardial infarction, coronary artery bypass graft, or percutaneous coronary intervention. Hemoglobin and high-sensitivity C-reactive protein (hsCRP) as well as glucose and hemoglobin A1c were analyzed at each university hospital laboratory according to standard methods. An hsCRP level above the clinical reference limit of 3 mg/L was considered increased.

Activity-related breathlessness was defined as a self-reported modified Medical Research Council score ≥2 (16, 17). Chronic bronchitis was defined as self-reported cough with phlegm for at least 3 consecutive months during at least 2 years. Wheeze was defined as an affirmative answer to the question “Do you have wheezing or whistling in your chest?”

Smoking history was retrieved from the questionnaires and categorized as current smokers, ex-smokers, and never-smokers. Ex-smokers were defined as those who had smoked for at least 1 year but not during the last year. Never-smokers were defined as those who gave an affirmative answer to the item “No, I have never smoked.” Pack-years were calculated for all participants with a history of smoking.

### Lung Function

Dynamic spirometry, including forced expiratory volume in one second (FEV_1_) and forced vital capacity (FVC), as well as diffusing capacity of the lung for carbon monoxide (Dl_CO_) tests_,_ were performed with the subject in the sitting position and wearing a nose clip, at least 15 minutes after inhalation of 400 μg of salbutamol, using a Jaeger Master Screen PFT (Vyaire). All procedures were performed in accordance with American Thoracic Society/European Respiratory Society standards (18, 19). In the present publication, CAL was defined according to GOLD as a post-bronchodilation FEV_1_/FVC < 0.7 (1), and all lung function data are presented using SCAPIS reference values (20). For comparison, CAL prevalence according to FEV_1_/FVC < LLN (21, 22) using both SCAPIS reference values (20) and Global Lung Function Initiative (GLI) (23) were calculated. Predicted values of GLI were calculated based on age, sex, and height (23) using the R package *rspiro* (24). Impaired Dl_CO_ was defined as a value below LLN (−1.64 standard deviation [SD]) using SCAPIS reference values (20).

### CT Scanning

All CT scanning was performed using a Somatom Definition Flash scanner (Siemens Healthcare), and the methodology has been described in detail (25, 26). CT scanners at each of the six sites used identical software, exam protocols, and hardware throughout the study, and the CT scans were read by trained readers at each site.

Emphysema was graded as none (0), mild (1; 1–25%), moderate (2; >25–50%), or severe (3; >50%) together with localization in the upper, middle, and/or lower part of right and/or left lung. The imaging terminology was based on suggested terminology by the Fleischner Society (27). Here, presence of emphysema was defined as CT findings of at least mild emphysema (grade 1) in any location. Emphysema was visually assessed as in the SCAPIS pilot study, where Krippendorff’s α was 0.8 for both inter- and intraobserver agreement of presence of emphysema (28). Furthermore, a total emphysema score was based on the sum of emphysema grade in the six different locations, leading to a sum ranging from 0 to 18.

Coronary artery calcifications (CACs) were assessed using electrocardiogram-gated noncontrast CT imaging at 120 kV as detailed elsewhere (29). A CAC score (CACS) > 100 (moderate or high calcification) was used as the cutoff value to define clinically relevant coronary artery calcifications in the present analyses.

### Statistical Analysis

Descriptive statistics, for the whole population and stratified by sex, were presented as numbers and percentages for categorical variables, whereas continuous variables were presented as mean and SD when symmetrically distributed and as median and interquartile range (IQR) otherwise. Airway symptoms and comorbidities were stratified by CAL, emphysema, and Dl_CO_, respectively, for total study population as well as for males and females separately. Venn diagrams illustrating the cooccurrence of CAL, emphysema, and impaired Dl_CO_ were created using the R package Biovenn (30).

Furthermore, six strata were formed based on participants’ CAL and smoking status. Prevalence ratios of breathlessness, chronic bronchitis, and wheeze between the strata were estimated using robust Poisson regression, adjusted for age, sex, and site. In accordance with recommendations for descriptive epidemiological studies (31), the number of covariates in the analyses were kept low to facilitate interpretation. The no-CAL/never-smoking group was used as reference. Robust Poisson regression models were also used to assess a possible statistical interaction between smoking and CAL status on the prevalence of breathlessness, chronic bronchitis, and wheeze, respectively. Corresponding analyses were also performed for emphysema and Dl_CO_ by smoking status strata. As a sensitivity analysis, analyses were reperformed excluding individuals with asthma, to eliminate the impact of asthma on CAL/emphysema.

Robust covariance matrix estimation for Poisson regressions were estimated from bootstrapping with 5,000 repeats using the robcov function from the rms package (32). When adjusted for, age and FVC were entered into models using restricted cubic splines with three knots, placed at the corresponding variable’s 10th, 50th, and 90th percentile. Statistical modeling was performed using R v4.2.2.

## Results

### Study Population

Study population characteristics by sex are given in Table 1. Individuals with complete information on smoking habits and lung function were included, comprising 28,746 adults (13,933 men [48%] and 14,813 women [52%]), of whom 14,558 were never-smokers (51%) and 14,188 (49%) were ever-smokers (10,500 ex-smokers [36%] and 3,688 current smokers [13%]).

The cumulative distribution of airflow obstruction in ex-smokers was more similar to never-smokers than to current smokers (*see* Figure E1 in the data supplement). Therefore, main analyses were performed on current smokers and ex-smokers separately. For comparison, data on never-smokers and ever-smokers (current plus ex-smokers) are presented as supplement tables (Tables E2–E4). Of note, among the ex-smokers, the median time since smoking cessation was 22 years (IQR, 12–30 years).

### CAL Prevalence

The prevalence of CAL in the whole study population was 8.8% (95% confidence interval [CI], 8.4–9.1%), with 19% (18–21%), 9.5% (9.0–10%), and 5.5% (5.2–5.9%) among current, ex-, and never-smokers, respectively. Overall, the CAL prevalence was 10% (9.7–11%) for men and 7.4% (7.0–7.8%) for women. When stratifying for sex, the CAL prevalence in current smokers was 22% (20–24%) in men and 17% (16–19%) in women, with the corresponding values for ex-smokers 11% (10–12%) versus 8.4% (7.7–9.2%) and for never-smokers 7.1% (6.6–7.7%) versus 3.8% (3.4–4.3%). Notably, as many as 32% of people with CAL were never-smokers. When participants with self-reported asthma were excluded, the prevalence of CAL was only slightly reduced, from 8.8% (8.4–9.1%) to 8.0% (7.7–8.4%), with the corresponding values in never-smokers from 5.5% (5.2–5.9%) to 4.9% (4.6–5.3%). Among never-smokers with CAL, 14% (11–16%) reported a history of asthma, and they had lower FEV_1_ and more respiratory symptoms than the subjects without asthma. There were more individuals with impaired Dl_CO_ in the subjects without asthma with CAL (Table 2). Among never-smokers without CAL, 4.8% had self-reported asthma, with lower proportions in the ex- and current smoking groups (Table 2).

**Table 2.** Symptoms and comorbidities in never-smoking participant with CAL with and without self-reported asthma

Characteristic	CAL/Asthma (*n* = *110*)	CAL/No Asthma (*n* = *668*)	*P* Value
Age, yr	57 (54–60)	58 (54–62)	0.076
Sex, men/women	42/68 (38/62)	223/443 (33/67)	0.3
Emphysema	6 (5.5)	33 (5.0)	0.8
Dl_CO_% predicted	106 (97–114)	100 (90–109)	<0.001
Impaired Dl_CO_	2 (1.9)	47 (7.2)	0.037
FEV_1_% predicted	82 (76–89)	88 (79–97)	<0.001
FEV_1_ < LLN	50 (45)	187 (28)	<0.001
Breathlessness	7 (6.4)	29 (4.4)	0.4
Chronic bronchitis	12 (11)	36 (5.5)	0.023
Wheeze	47 (43)	56 (8.5)	<0.001
Ischemic heart disease	3 (2.7)	17 (2.5)	0.8
CACS > 100	17 (16)	113 (17)	0.8
Diabetes mellitus	9 (8.2)	34 (5.1)	0.2
hsCRP > 3 mg/L	15 (14)	89 (13)	>0.9

*Definition of abbreviations*: CACS = coronary artery calcifications score; CAL = chronic airflow limitation; Dl_CO_ = diffusing capacity of the lung for carbon monoxide; FEV_1_ = forced expiratory volume in 1 second; hsCRP = high-sensitivity C-reactive protein; LLN = lower limit of normal.

Data are given as median (interquartile range) or *n* (%). P value determined by Wilcoxon rank-sum test, Pearson’s chi-square test, or Fisher’s exact test.

**Figure 2.**
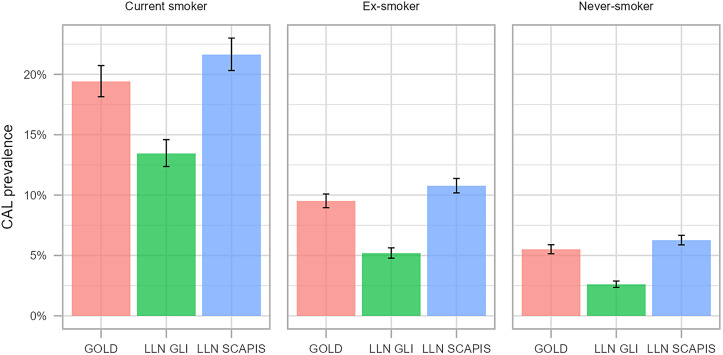
Prevalence of CAL with 95% confidence intervals by smoking status, according to GOLD, GLI < LLN, and SCAPIS < LLN. CAL = chronic airflow limitation; GLI = Global Lung Function Initiative; GOLD = Global Initiative for Obstructive Lung Disease; LLN = lower limit of normal; SCAPIS = Swedish Cardiopulmonary Bioimage Study.

The prevalence of CAL differed depending on the definition used and was 4.9% (4.7–5.2%) and 9.9% (9.5–10%) in the whole study population, when defined as FEV_1_/FVC < GLI-LLN and <SCAPIS-LLN respectively (Figure 2).

### Emphysema Prevalence

In total, 404 individuals (1.4%) had missing emphysema data, mainly due to not performing the CT scan (*n* = 372). Prevalence of emphysema in the whole study population was 5.7% (95% CI, 5.5–6.0%) and was higher in current smokers (18% [17–20%] than ex-smokers (6.5% [6.0–7.0%]) and never-smokers (2.1% [1.9–2.3%]). The prevalence was higher for males (6.3% [5.9–6.7%]) than for females (5.2% [4.8–5.6%]). Among those with emphysema, 18% were never-smokers. Exclusion of self-reported asthma did not alter the emphysema prevalence. In Figure E2, the distribution of emphysema scores is presented for each of the smoking status groups.

### Prevalence of Impaired Dl_CO_

In total, 847 (2.9%) individuals had missing data on Dl_CO_. Prevalence of impaired Dl_CO_ in the whole study population was 8.8% (95% CI, 8.5–9.2%) and was not altered when individuals with self-reported asthma were excluded (8.9% [8.5–9.2%]). The prevalence was higher in current smokers (25% [24–26%]) compared with ex-smokers (8.1% [7.5–8.6%]) and never-smokers (5.3% [5.0–5.7%]). The prevalence was lower for males (8.3% [7.8–8.8%]) than for females (9.4% [8.9–9.9%]). Among those with impaired Dl_CO_, 31% were never-smokers.

### Cooccurrence of CAL, Emphysema, and Impaired Dl_CO_

**Figure 3.**
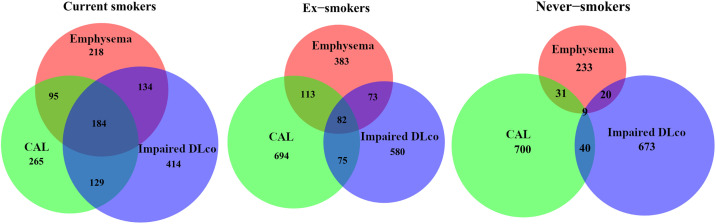
Venn diagram illustrating the cooccurrence of chronic airflow limitation (CAL), emphysema, and impaired diffusing capacity of the lung for carbon monoxide (Dl_CO_) in (*A*) current smokers (*n* = 1,439), (*B*) ex-smokers (*n* = 2,000), and (*C*) never-smokers (*n* = 1,706), respectively. Note that the circles are proportional to the prevalence of CAL, emphysema, and impaired Dl_CO_ within, but not between, smoking status groups.

Cooccurrence of CAL, emphysema, and impaired Dl_CO_ was markedly more frequent in current smokers than in never-smokers, with ex-smokers in between (Figure 3). Among current smokers with CAL, 27% also had both emphysema and impaired Dl_CO_, whereas only 1.2% of the never-smokers had all these three findings. Notably, emphysema was clearly less prevalent in never-smokers (2.1%) compared with CAL (5.5%) and impaired Dl_CO_ (5.3%). A similar pattern, yet not as clear, was seen when comparing ever-smokers and never-smokers (Figure E3). Data on site-specific prevalence of CAL, emphysema, and Dl_CO_ indicate site variability (Table E4).

### Respiratory Symptoms and Comorbidities by CAL, Emphysema, Impaired Dl_CO_, and Smoking Status

In general, a history of smoking was associated with an increased prevalence of airway symptoms and comorbidities (Tables 3–5). Respiratory symptoms, ischemic heart disease, and diabetes were more prevalent among individuals with CAL and impaired Dl_CO_ irrespective of smoking status but higher in current smokers. This pattern was not seen among never-smoking individuals with emphysema. Self-reported asthma was more common in individuals with CAL, regardless of smoking habits, and was more prevalent in never-smokers. The prevalence of breathlessness in never-smokers was almost three times as frequent among women as men, whereas no sex differences were seen for lung function (Tables E5–E10).

**Table 3.** Lung physiology, airway symptoms, and comorbidities by CAL and smoking status

	No CAL			CAL		
	Never-Smoker (*n* = 13,755)	Ex-smoker (*n* = 9,501)	Current Smoker (*n* = 2,972)	Never-Smoker (*n* = 803)	Ex-smoker (*n* = 999)	Current Smoker (*n* = 716)
Emphysema	261 (1.9)	471 (5.0)	368 (13)	40 (5.0)	200 (20)	289 (42)
Dl_CO_% predicted	99 (91–108)	98 (89–107)	91 (82–101)	101 (91–110)	94 (83–105)	81 (70–93)
Dl_CO_ < LLN	705 (5.3)	662 (7.2)	563 (20)	51 (6.5)	163 (17)	323 (47)
FEV_1_% predicted	100 (92–108)	99 (91–107)	96 (88–105)	87 (78–96)	84 (75–92)	79 (68–89)
FEV_1_ < LLN	739 (5.4)	544 (5.7)	311 (10)	252 (31)	385 (39)	382 (53)
Breathlessness	440 (3.2)	430 (4.5)	161 (5.5)	40 (5.0)	104 (10)	107 (15)
Chronic bronchitis	475 (3.5)	408 (4.4)	229 (8.0)	56 (7.1)	98 (10)	131 (19)
Wheeze	561 (4.1)	573 (6.1)	435 (15)	110 (14)	166 (17)	241 (35)
Asthma	651 (4.8)	429 (4.6)	103 (3.6)	110 (14)	113 (12)	55 (8.0)
Ischemic heart disease	238 (1.7)	279 (2.9)	77 (2.6)	21 (2.6)	54 (5.4)	37 (5.2)
CACS > 100	2,019 (15)	2,000 (22)	730 (25)	135 (17)	298 (32)	256 (38)
Diabetes mellitus	845 (6.1)	776 (8.2)	272 (9.2)	45 (5.6)	83 (8.3)	86 (12)
hsCRP > 3 mg/L	1,908 (14)	1,640 (17)	623 (21)	110 (14)	212 (21)	191 (27)
Pack-years of cigarette smoking	—	9.8 (4.2–18)	20 (11–32)	—	15 (6.0–26)	32 (20–39)

*Definition of abbreviations*: CACS = coronary artery calcifications score; CAL = chronic airflow limitation; Dl_CO_ = diffusing capacity of the lung for carbon monoxide; FEV_1_ = forced expiratory volume in 1 second; hsCRP = high-sensitivity C-reactive protein; LLN = lower limit of normal.

Data are given as median (interquartile range) or *n* (%).

**Table 4.** Lung physiology, airway symptoms, and comorbidities by emphysema and smoking status

	No Emphysema			Emphysema		
	Never-Smoker (*n* = 14,068)	Ex-smoker (*n* = 9,683)	Current Smoker (*n* = 2,962)	Never-Smoker (*n* = 301)	Ex-smoker (*n* = 671)	Current Smoker (*n* = 657)
CAL	755 (5.4)	783 (8.1)	406 (14)	40 (13)	200 (30)	289 (44)
Dl_CO_% predicted	99 (91–108)	98 (89–107)	92 (82–101)	97 (87–106)	91 (80–101)	79 (68–90)
Dl_CO_ < LLN	713 (5.2)	655 (6.9)	543 (19)	29 (9.9)	155 (24)	318 (50)
FEV_1_% predicted	99 (91–107)	98 (90–107)	95 (85–104)	97 (88–106)	93 (82–103)	88 (76–98)
FEV_1_ < LLN	936 (6.7)	767 (7.9)	454 (15)	34 (11)	141 (21)	219 (33)
Breathlessness	459 (3.3)	460 (4.8)	180 (6.1)	8 (2.7)	60 (9.0)	76 (12)
Chronic bronchitis	511 (3.7)	442 (4.7)	259 (9.1)	10 (3.4)	56 (8.5)	94 (15)
Wheeze	650 (4.7)	655 (6.9)	504 (18)	15 (5.0)	68 (10)	151 (24)
Asthma	729 (5.3)	493 (5.2)	127 (4.4)	19 (6.4)	42 (6.4)	27 (4.3)
Ischemic heart disease	249 (1.8)	292 (3.0)	82 (2.8)	9 (3.0)	38 (5.7)	27 (4.1)
CACS > 100	2,097 (15)	2,085 (22)	748 (26)	55 (19)	210 (33)	236 (37)
Diabetes mellitus	867 (6.2)	785 (8.1)	292 (9.9)	13 (4.3)	59 (8.8)	60 (9.1)
hsCRP > 3 mg/L	1,949 (14)	1,667 (17)	613 (21)	46 (15)	155 (23)	181 (28)
Pack-years of cigarette smoking	—	9.5 (4.0–18)	20 (11–32)	—	21 (12–32)	30 (20–38)

*Definition of abbreviations*: CACS = coronary artery calcifications score; CAL = chronic airflow limitation; Dl_CO_ = diffusing capacity of the lung for carbon monoxide; FEV_1_ = forced expiratory volume in 1 second; hsCRP = high-sensitivity C-reactive protein; LLN = lower limit of normal.

Data are given as median (interquartile range) or *n* (%).

**Table 5.** Lung physiology, airway symptoms, and comorbidities by diffusing capacity for carbon monoxide and smoking status

	Normal Dl_CO_			Impaired Dl_CO_		
	Never-Smoker (*n* = 13,380)	Ex-smoker (*n* = 9,397)	Current Smoker (*n* = 2,655)	Never-Smoker (*n* = 756)	Ex-smoker (*n* = 825)	Current Smoker (*n* = 886)
CAL	737 (5.5)	814 (8.7)	370 (14)	51 (6.7)	163 (20)	323 (36)
Emphysema	264 (2.0)	496 (5.3)	313 (12)	29 (3.9)	155 (19)	318 (37)
FEV_1_% predicted	100 (92–108)	99 (90–107)	96 (87–105)	90 (81–99)	88 (78–96)	84 (74–94)
FEV_1_ < LLN	765 (5.7)	652 (6.9)	312 (12)	183 (24)	242 (29)	346 (39)
Breathlessness	398 (3.0)	401 (4.3)	137 (5.2)	62 (8.3)	114 (14)	118 (14)
Chronic bronchitis	489 (3.7)	434 (4.7)	209 (8.2)	31 (4.2)	58 (7.2)	128 (15)
Wheeze	597 (4.5)	625 (6.7)	409 (16)	56 (7.5)	93 (12)	227 (27)
Asthma	718 (5.5)	488 (5.3)	113 (4.4)	25 (3.4)	41 (5.1)	40 (4.7)
Ischemic heart disease	212 (1.6)	250 (2.7)	60 (2.3)	32 (4.2)	62 (7.5)	49 (5.5)
CACS > 100	1,977 (15)	1,992 (22)	638 (25)	119 (17)	236 (30)	296 (35)
Diabetes mellitus	783 (5.9)	723 (7.7)	237 (8.9)	75 (9.9)	109 (13)	107 (12)
hsCRP > 3 mg/L	1,754 (13)	1,584 (17)	493 (19)	783 (5.9)	723 (7.7)	237 (8.9)
Pack-years of cigarette smoking	—	9.8 (4.0–18)	20 (10–32)	—	16 (7.0–27)	29 (19–38)

*Definition of abbreviations*: CACS = coronary artery calcifications score; CAL = chronic airflow limitation; Dl_CO_ = diffusing capacity of the lung for carbon monoxide; FEV_1_ = forced expiratory volume in 1 second; hsCRP = high-sensitivity C-reactive protein; LLN = lower limit of normal.

Data are given as median (interquartile range) or *n* (%).

**Figure 4.**
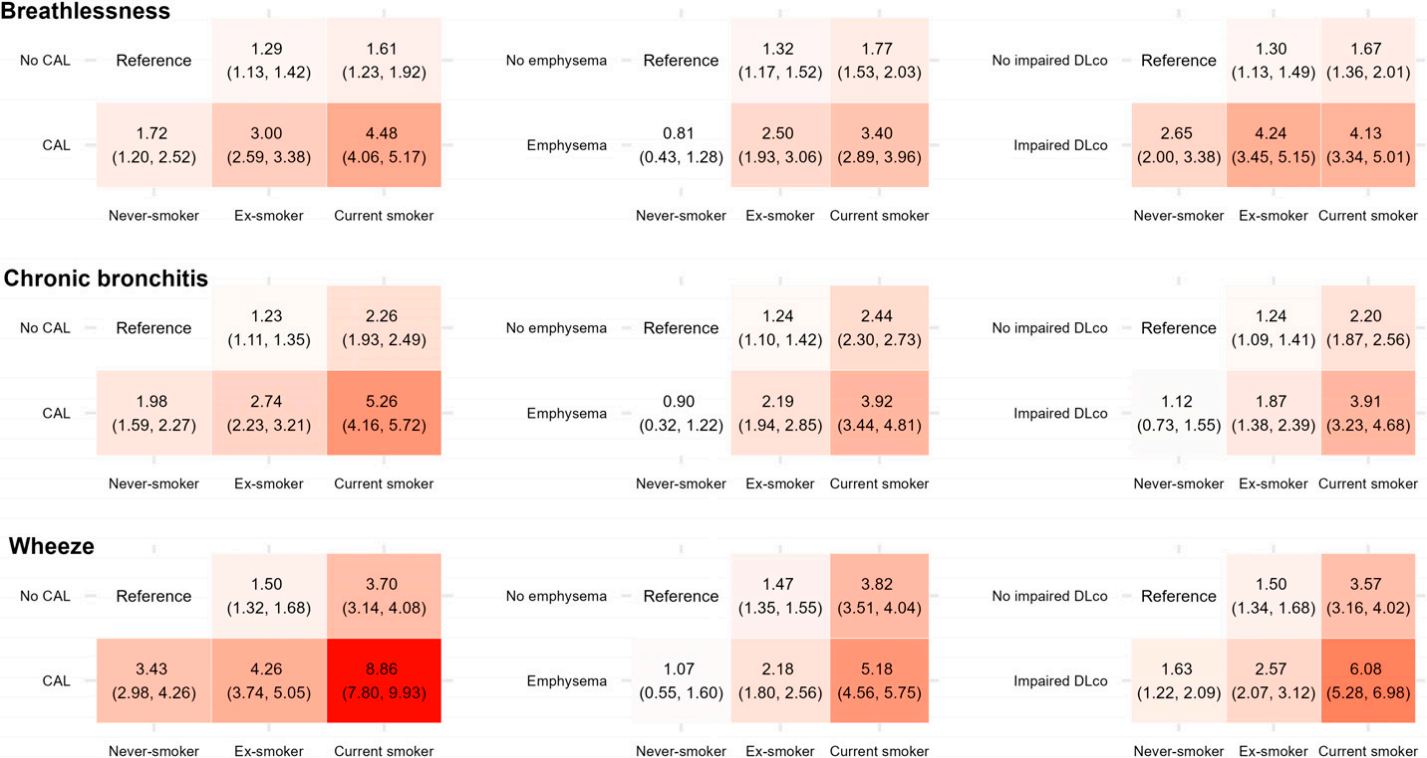
Prevalence ratios for respiratory symptoms (breathlessness [upper panel], chronic bronchitis [middle panel], and wheeze [lower panel]) by CAL and smoking status (left panel), by emphysema and smoking status (middle panel), and by impaired Dl_CO_ and smoking status (right panel). Red color intensity is proportional to magnitude of prevalence ratio. The ratios are adjusted for sex, age, and site. CAL = chronic airflow limitation; Dl_CO_ = diffusing capacity of the lung for carbon monoxide.

Both smoking burden and CAL were associated with an increase in respiratory symptoms (Figure 4). The prevalence of respiratory symptoms among never-smoking individuals with CAL and current smoking individuals without CAL were comparable. Among never-smokers, in contrast to current and former smokers, respiratory symptoms were similar regardless of emphysema (Figure 4). The picture was somewhat different concerning Dl_CO_. Among never-smokers, the prevalence of breathlessness and wheeze was clearly increased in never-smokers with impaired Dl_CO_, whereas the presence of chronic bronchitis was not. Unadjusted prevalence ratios were similar (Figure E4). When excluding participants with asthma from the analyses, the adjusted prevalence ratios of respiratory symptoms did not substantially change in individuals with either CAL or emphysema, regardless of smoking habits (Figure E5).

Analysis of statistical interactions showed that the association between CAL and breathlessness was significantly stronger among current smokers than never-smokers (*P* = 0.005), whereas the association between wheeze and CAL was weaker (*P* = 0.006). For chronic bronchitis, no significant interaction was found (*P* = 0.33). Details of interaction analyses regarding CAL, emphysema, and Dl_CO_ are presented in the data supplement (Tables E11–E13).

## Discussion

This large, random, population-based investigation of middle-aged men and women, with approximately 50% never-smokers, enabled a unique possibility to study the prevalence of CAL, emphysema, and impaired Dl_CO_ in relation to smoking status and respiratory symptoms. The overall prevalence of CAL, emphysema, and impaired Dl_CO_ was 8.8%, 5.7%, and 8.9%, respectively, but highly dependent on smoking history. Among never-smokers, the CAL prevalence was 5.5%. As many as approximately one-third of the individuals with CAL and impaired Dl_CO_ and one-fifth of those with emphysema had never smoked. Regardless of smoking status, individuals with CAL and impaired Dl_CO_ reported more breathlessness, chronic bronchitis, and wheeze compared with those without CAL. A history of asthma was reported in 14% of never-smokers with CAL and associated with a higher prevalence of respiratory symptoms.

The prevalence of CAL was slightly lower than in a previous study (3), in which individuals within the same age range were included but with a selection based on social economy. Depending on the age range included, similar or higher prevalence ratios have been reported (33–35). In general, the CAL prevalence in the current study was low compared with international data (5, 6, 35, 36). This may be explained by the low smoking prevalence in Sweden (from an international point of view), 6% according to the Public Health Agency of Sweden (37), as well as the limited upper age range of 64 years in this study. Furthermore, when comparing the FEV_1_/FVC ratio between groups with different smoking history, ex-smokers were found to be more similar to never-smokers. This is probably because of a higher historical smoking burden, as current smokers had smoked approximately twice as much compared with ex-smokers. Furthermore, time since smoking cessation in ex-smokers was long (median, 22 yr). These circumstances motivated us to divide ever-smokers in the primary analysis into current smokers and ex-smokers to enlighten the impact of active smoking in the analyses.

In the present study, the prevalence of CAL, as stated by the GOLD definition of COPD (i.e., FEV_1_/FVC < 0.7), was compared with the internationally frequently used definition of CAL according to GLI LLN (23) as well as with the local SCAPIS LLN (20). Prevalence of CAL according to the fixed ratio and SCAPIS LLN was similar within this age interval of 50–64 years, whereas it was evident that the prevalence of CAL using the GLI LLN was markedly lower. Other studies have also shown that GLI LLN is not the optimal criterion to define CAL in Swedish middle-aged individuals (38). Similar Danish data showed a prevalence of airflow limitation ranging from 8% to 17% when GOLD and four different LLN criteria were compared. Yet, the risk of COPD exacerbations and mortality was similar among the different criteria. It was further emphasized that local LLN criteria would be more optimal to identify high-risk individuals (39), thus supporting the effort within the SCAPIS study to develop local spirometric reference values (20).

One-third of the individuals with CAL were never-smokers, highlighting the fact that CAL is not uncommon among Swedish never-smokers within this age range. This proportion is similar to what was found in previous studies, where never-smokers in a CAL population have been found to constitute around 20–50% (10–12). Here, approximately 14% of never-smokers with CAL had a history of self-reported asthma, which is low compared with a Danish study in which a corresponding prevalence of 28% was found (40), however, using CAL based on prebronchodilator values and without restriction on age at diagnosis. As in the present study, patients with asthma with CAL in the study by Çolak and colleagues (40) had lower FEV_1 _and experienced more respiratory symptoms than those with CAL but no asthma. Importantly, when never-smoking participants with self-reported asthma were excluded in the present analysis, the prevalence of CAL was only marginally lower, implying that factors other than asthma may be important for development of CAL, as also suggested by others (40, 41). This may include factors such as early-life events, including prematurity, viral airway infections, and air pollution (12, 13). Although being less obstructive than smokers with CAL, the data show that important respiratory symptoms, such as breathlessness, chronic bronchitis, and wheeze, were 1.5 to 3 times more common in never-smokers with CAL compared with never-smokers without CAL, and these differences persisted after adjustment for age, sex, and site.

The overall prevalence of emphysema was 5.6% and was considerably lower in never-smokers (2.1%). Cooccurrence of CAL and emphysema was low among never-smokers, suggesting that airflow obstruction in never-smokers is mainly located in the airways and not in the lung parenchyma. This supports the hypothesis about different pathophysiological mechanisms behind CAL in never-smokers compared with ever-smokers and, thus, implying different clinical phenotypes. Population-based data on emphysema are scarce, but even though a liberal definition of “any emphysema” on CT was used, the proportion of individuals with emphysema was low in relation to international data (8). Importantly, when addressing the severity of emphysema, as determined by emphysema score (Figure E2), the prevalence of moderate or severe emphysema was very low, probably because of the low smoking burden and low occupational exposure in Sweden in general.

In this population, the prevalence of impaired Dl_CO_ was 8.8% and was lower in never-smokers (5.3%). Of note, as for CAL, almost one-third of the individuals with impaired Dl_CO_ were never-smokers and had more respiratory symptoms, mainly breathlessness. This group also had a higher prevalence of emphysema and CAL as well as self-reported ischemic heart disease and diabetes, compared with never-smokers with normal Dl_CO_. Thus, it is possible that respiratory factors other than respiratory may affect Dl_CO_. For instance, diabetes has previously been shown to reduce both lung function and Dl_CO _(42).

The prevalence of breathlessness, chronic bronchitis, and wheeze in current smoking individuals without CAL and never-smoking individuals with CAL was comparable, indicating that respiratory symptoms are common among smokers, even without any signs of airflow obstruction after bronchodilation. The higher prevalence of breathlessness in women regardless of smoking habits is in line with previous data and may at least partly be explained by the lower absolute FVC values in women (17). Among smokers without CAL, reduced Dl_CO _and presence of emphysema were two to three times more common than among never-smokers with CAL. This implies that despite signs of alveolar destruction, in terms of emphysema and impaired diffusing capacity, an FEV_1_/FVC < 0.70 does not detect all tobacco-related lung injuries. Yet, it should be noted that the magnitude of reduction in Dl_CO_ and prevalence of moderate to severe emphysema also in ex-smokers and current smokers were low in this population.

Interaction analysis showed that the association between CAL and breathlessness was stronger among smokers, whereas the association between CAL and wheeze was stronger in never-smokers. Among people with CAL, these interactions may be related to many factors, as impaired Dl_CO_, emphysema, cardiovascular comorbidities, diabetes, and increased hsCRP were more common among current smokers, whereas asthma was more prevalent among never-smoking individuals.

### Strengths and Limitations

The strength of SCAPIS is the large general population-based sample, in which 28,746 subjects with data on smoking habits have undergone CT imaging and spirometry, including Dl_CO_, after bronchodilation. The large proportion of never-smokers and the relatively high prevalence of never-smokers with CAL also made it possible to address differences in common respiratory symptoms and comorbidities in CAL related to smoking habits. The findings in never-smokers with CAL also persisted when self-reported asthma was excluded. Although a limitation is the restricted age group investigated (50–64 yr), it is known that CAL often makes its debut during middle age. Other limitations are that spirometry was only performed once, something that may reduce the reproducibility of the lung function data, and that comorbidities were based on questionnaires with self-reported data. Dl_CO_ data were not corrected for hemoglobin levels, but this is considered a minor issue, as average hemoglobin values were well within normal values. Finally, the study was performed as a descriptive cross-sectional analysis and, thus, causality cannot be addressed.

### Conclusions

In this large population-based study of middle-aged people, CAL and impaired Dl_CO_ were associated with common respiratory symptoms regardless of smoking habits. The findings suggest that CAL in never-smokers signifies a separate clinical phenotype that may be monitored and, possibly, treated differently from smoking-related COPD.

## Supplemental Materials

10.1513/AnnalsATS.202402-122OC Online Data Supplement

## References

[1] Agustí A Celli BR Criner GJ Halpin D Anzueto A Barnes P *et al.* Global Initiative for Chronic Obstructive Lung Disease 2023 report: GOLD executive summary *Am J Respir Crit Care Med* 2023 207 819 837 36856433 10.1164/rccm.202301-0106PP PMC10111975

[2] Çolak Y Afzal S Nordestgaard BG Vestbo J Lange P Prognosis of asymptomatic and symptomatic, undiagnosed COPD in the general population in Denmark: a prospective cohort study *Lancet Respir Med* 2017 5 426 434 28389225 10.1016/S2213-2600(17)30119-4

[3] Torén K Olin AC Lindberg A Vikgren J Schiöler L Brandberg J *et al.* Vital capacity and COPD: the Swedish CArdioPulmonary bioImage Study (SCAPIS) *Int J Chron Obstruct Pulmon Dis* 2016 11 927 933 27194908 10.2147/COPD.S104644 PMC4859418

[4] Brisman J Kim JL Olin AC Torén K Bake B Spirometric reference equations for Swedish adults *Clin Physiol Funct Imaging* 2017 37 640 645 26865107 10.1111/cpf.12349

[5] Buist AS McBurnie MA Vollmer WM Gillespie S Burney P Mannino DM *et al.* BOLD Collaborative Research Group International variation in the prevalence of COPD (the BOLD Study): a population-based prevalence study *Lancet* 2007 370 741 750 17765523 10.1016/S0140-6736(07)61377-4

[6] Menezes AM Perez-Padilla R Jardim JR Muiño A Lopez MV Valdivia G *et al.* PLATINO Team Chronic obstructive pulmonary disease in five Latin American cities (the PLATINO study): a prevalence study *Lancet* 2005 366 1875 1881 16310554 10.1016/S0140-6736(05)67632-5

[7] Woodruff PG Barr RG Bleecker E Christenson SA Couper D Curtis JL *et al.* SPIROMICS Research Group Clinical significance of symptoms in smokers with preserved pulmonary function *N Engl J Med* 2016 374 1811 1821 27168432 10.1056/NEJMoa1505971 PMC4968204

[8] Tan WC Hague CJ Leipsic J Bourbeau J Zheng L Li PZ *et al.* Canadian Respiratory Research Network and the CanCOLD Collaborative Research group Findings on thoracic computed tomography scans and respiratory outcomes in persons with and without chronic obstructive pulmonary disease: a population-based cohort study *PLoS One* 2016 11 e0166745 27861566 10.1371/journal.pone.0166745 PMC5115801

[9] Tan WC Sin DD Bourbeau J Hernandez P Chapman KR Cowie R *et al.* CanCOLD Collaborative Research Group Characteristics of COPD in never-smokers and ever-smokers in the general population: results from the CanCOLD study *Thorax* 2015 70 822 829 26048404 10.1136/thoraxjnl-2015-206938

[10] Lamprecht B Schirnhofer L Kaiser B Buist S Studnicka M Non-reversible airway obstruction in never smokers: results from the Austrian BOLD study *Respir Med* 2008 102 1833 1838 18722100 10.1016/j.rmed.2008.07.007

[11] Lamprecht B McBurnie MA Vollmer WM Gudmundsson G Welte T Nizankowska-Mogilnicka E *et al.* BOLD Collaborative Research Group COPD in never smokers: results from the population-based burden of obstructive lung disease study *Chest* 2011 139 752 763 20884729 10.1378/chest.10-1253 PMC3168866

[12] Yang IA Jenkins CR Salvi SS Chronic obstructive pulmonary disease in never-smokers: risk factors, pathogenesis, and implications for prevention and treatment *Lancet Respir Med* 2022 10 497 511 35427530 10.1016/S2213-2600(21)00506-3

[13] Pando-Sandoval A Ruano-Ravina A Candal-Pedreira C Rodríguez-García C Represas-Represas C Golpe R *et al.* Risk factors for chronic obstructive pulmonary disease in never-smokers: a systematic review *Clin Respir J* 2022 16 261 275 35142054 10.1111/crj.13479 PMC9060104

[14] Bergström G Berglund G Blomberg A Brandberg J Engström G Engvall J *et al.* The Swedish CArdioPulmonary BioImage Study: objectives and design *J Intern Med* 2015 278 645 659 26096600 10.1111/joim.12384 PMC4744991

[15] Torén K Brisman J Järvholm B Asthma and asthma-like symptoms in adults assessed by questionnaires: a literature review *Chest* 1993 104 600 608 7802735 10.1378/chest.104.2.600

[16] Bestall JC Paul EA Garrod R Garnham R Jones PW Wedzicha JA Usefulness of the Medical Research Council (MRC) dyspnoea scale as a measure of disability in patients with chronic obstructive pulmonary disease *Thorax* 1999 54 581 586 10377201 10.1136/thx.54.7.581 PMC1745516

[17] Ekström MP Blomberg A Bergström G Brandberg J Caidahl K Engström G *et al.* The association of body mass index, weight gain and central obesity with activity-related breathlessness: the Swedish Cardiopulmonary Bioimage Study *Thorax* 2019 74 958 964 31434752 10.1136/thoraxjnl-2019-213349

[18] Miller MR Hankinson J Brusasco V Burgos F Casaburi R Coates A *et al.* ATS/ERS Task Force Standardisation of spirometry *Eur Respir J* 2005 26 319 338 16055882 10.1183/09031936.05.00034805

[19] Graham BL Brusasco V Burgos F Cooper BG Jensen R Kendrick A *et al.* 2017 ERS/ATS standards for single-breath carbon monoxide uptake in the lung *Eur Respir J* 2017 49 1600016 28049168 10.1183/13993003.00016-2016

[20] Malinovschi A Xingwu Z Andersson A Backman H Bake B Blomberg A *et al.* Consequences of using post- or pre-bronchodilatory reference values in interpreting spirometry *Am J Respir Crit Care Med* 2023 208 461 471 37339507 10.1164/rccm.202212-2341OC

[21] Pellegrino R Viegi G Brusasco V Crapo RO Burgos F Casaburi R *et al.* Interpretative strategies for lung function tests *Eur Respir J* 2005 26 948 968 16264058 10.1183/09031936.05.00035205

[22] Vaz Fragoso CA Concato J McAvay G van Ness PH Rochester CL Yaggi HK *et al.* The ratio of FEV_1_ to FVC as a basis for establishing chronic obstructive pulmonary disease *Am J Respir Crit Care Med* 2010 181 446 451 20019341 10.1164/rccm.200909-1366OC PMC3159085

[23] Quanjer PH Stanojevic S Cole TJ Baur X Hall GL Culver BH *et al.* ERS Global Lung Function Initiative Multi-ethnic reference values for spirometry for the 3-95-yr age range: the global lung function 2012 equations *Eur Respir J* 2012 40 1324 1343 22743675 10.1183/09031936.00080312 PMC3786581

[24] Lytras T 2020 https://CRAN.R-project.org/package=rspiro

[25] Lynch DA Austin JH Hogg JC Grenier PA Kauczor HU Bankier AA *et al.* CT-definable subtypes of chronic obstructive pulmonary disease: a statement of the Fleischner Society *Radiology* 2015 277 192 205 25961632 10.1148/radiol.2015141579 PMC4613878

[26] Toren K Vikgren J Olin AC Rosengren A Bergstrom G Brandberg J Occupational exposure to vapor, gas, dust, or fumes and chronic airflow limitation, COPD, and emphysema: the Swedish CArdioPulmonary BioImage Study (SCAPIS pilot) *Int J Chron Obstruct Pulmon Dis* 2017 12 3407 3413 29238185 10.2147/COPD.S144933 PMC5713698

[27] Hansell DM Bankier AA MacMahon H McLoud TC Muller NL Remy J Fleischner Society: glossary of terms for thoracic imaging *Radiology* 2008 246 697 722 18195376 10.1148/radiol.2462070712

[28] Vikgren J Khalil M Cederlund K Sörensen K Boijsen M Brandberg J *et al.* Visual and quantitative evaluation of emphysema: a case-control study of 1111 participants in the pilot Swedish CArdioPulmonary BioImage Study (SCAPIS) *Acad Radiol* 2020 27 636 643 31326310 10.1016/j.acra.2019.06.019

[29] Bergström G Persson M Adiels M Björnson E Bonander C Ahlström H *et al.* Prevalence of subclinical coronary artery atherosclerosis in the general population *Circulation* 2021 144 916 929 34543072 10.1161/CIRCULATIONAHA.121.055340 PMC8448414

[30] Hulsen T BioVenn: an R and Python package for the comparison and visualization of biological lists using area-proportional Venn diagrams *Data Sci* 2021 4 51 61 10.1186/1471-2164-9-488 PMC2584113 18925949

[31] Lesko CR Fox MP Edwards JK A framework for descriptive epidemiology *Am J Epidemiol* 2022 191 2063 2070 35774001 10.1093/aje/kwac115 PMC10144679

[32] Harrell FE Jr. 2021 cran.r-project.org/web/packages/rms

[33] Backman H Vanfleteren L Lindberg A Ekerljung L Stridsman C Axelsson M *et al.* Decreased COPD prevalence in Sweden after decades of decrease in smoking *Respir Res* 2020 21 283 33115506 10.1186/s12931-020-01536-4 PMC7594463

[34] Danielsson P Ólafsdóttir IS Benediktsdóttir B Gíslason T Janson C The prevalence of chronic obstructive pulmonary disease in Uppsala, Sweden—the Burden of Obstructive Lung Disease (BOLD) study: cross-sectional population-based study *Clin Respir J* 2012 6 120 127 21651748 10.1111/j.1752-699X.2011.00257.x

[35] Fabricius P Løkke A Marott JL Vestbo J Lange P Prevalence of COPD in Copenhagen *Respir Med* 2011 105 410 417 20952174 10.1016/j.rmed.2010.09.019

[36] Tan WC Bourbeau J FitzGerald JM Cowie R Chapman K Hernandez P *et al.* Can age and sex explain the variation in COPD rates across large urban cities? A population study in Canada *Int J Tuberc Lung Dis* 2011 15 1691 1698 22118181 10.5588/ijtld.11.0211

[37] Public Health Agency of Sweden 2021 https://www.folkhalsomyndigheten.se/the-public-health-agency-of-sweden/living-conditions-and-lifestyle/andtg/tobacco/

[38] Malinovschi A Zhou X Bake B Bergström G Blomberg A Brisman J *et al.* Assessment of Global Lung Function Initiative (GLI) reference equations for diffusing capacity in relation to respiratory burden in the Swedish CArdioPulmonary bioImage Study (SCAPIS) *Eur Respir J* 2020 56 1901995 32341107 10.1183/13993003.01995-2019

[39] Çolak Y Nordestgaard BG Vestbo J Lange P Afzal S Comparison of five major airflow limitation criteria to identify high-risk individuals with COPD: a contemporary population-based cohort *Thorax* 2020 75 944 954 32820083 10.1136/thoraxjnl-2020-214559

[40] Çolak Y Afzal S Nordestgaard BG Lange P Majority of never-smokers with airflow limitation do not have asthma: the Copenhagen General Population Study *Thorax* 2016 71 614 623 27015799 10.1136/thoraxjnl-2015-208178

[41] Thomsen M Nordestgaard BG Vestbo J Lange P Characteristics and outcomes of chronic obstructive pulmonary disease in never smokers in Denmark: a prospective population study *Lancet Respir Med* 2013 1 543 550 24461615 10.1016/S2213-2600(13)70137-1

[42] Anandhalakshmi S Manikandan S Ganeshkumar P Ramachandran C Alveolar gas exchange and pulmonary functions in patients with type II diabetes mellitus *J Clin Diagn Res* 2013 7 1874 1877 24179886 10.7860/JCDR/2013/6550.3339 PMC3809625

