# Supplementary material for: Chronic Airflow Limitation, Emphysema, and Impaired Diffusing Capacity in Relation to Smoking Habits in a Swedish Middle-aged Population
Source: Ann Am Thorac Soc. 2024 Dec 1;21(12):1678–87. doi: 10.1513/AnnalsATS.202402-122OC (PMC11622819; doi:10.1513/AnnalsATS.202402-122OC)
Supplement: Online Data Supplement [file AnnalsATS.202402-122OCS1.docx]

**Chronic Airflow Limitation, Emphysema and Impaired Diffusing Capacity in Relation to Smoking Habits in a Swedish Middle-Aged Population**

Anders Blomberg, Kjell Torén, Per Liv, Gabriel Granåsen, Anders Andersson, Annelie Behndig, Göran Bergström, John Brandberg, Kenneth Caidahl, Kerstin Cederlund, Arne Egesten^l^, Magnus Ekström, Maria J Eriksson, Emil Hagström, Christer Janson, Tomas Jernberg, David Kylhammar, Lars Lind, Anne Lindberg, Eva Lindberg, Claes-Göran Löfdahl, Andrei Malinovschi, Maria Mannila, Lars T Nilsson, Anna-Carin Olin, Anders Persson, Hans Lennart Persson, Annika Rosengren, Johan Sundström, Eva Swahn, Stefan Söderberg, Jenny Vikgren, Per Wollmer, Carl Johan Östgren, Jan Engvall, C. Magnus Sköld

**ONLINE DATA SUPPLEMENT**

# SCAPIS Study Organization

## Study governance (active and former)

### National Steering Committee

#### Directors:

Göran Bergström, University of Gothenburg, Director

Carl Johan Östgren, Linköping University, vice Director

#### Members (active and former):

Anders Blomberg, Umeå University, Umeå; John Brandberg, University of Gothenburg, Gothenburg; Kerstin Cederlund, Karolinska Institutet, Stockholm; Gunnar Engström, Lund University, Lund; Jan Engvall, Linköping University, Linköping; Mats G. Hansson, Uppsala University, Uppsala; Tomas Jernberg, Karolinska Institutet, Stockholm; Lars Lind, Uppsala University, Uppsala; Eva Lindberg,Uppsala University, Uppsala; Margaretha Persson, Lund University, Lund; Fredrik Nyström, Linköping University, Linköping; Annika Rosengren, University of Gothenburg, Gothenburg; Magnus Sköld,Karolinska Institutet, Stockholm; Johan Sundström, Uppsala University, Uppsala; Stefan Söderberg,Umeå University, Umeå; Kjell Torén, University of Gothenburg, Gothenburg

#### Coordinator:

Bim Boberg, the Swedish Heart and Lung Foundation, Stockholm

The Swedish CArdioPulmonary bioImage Study (SCAPIS) is a collaborative project between the following

Swedish Universities and Swedish University Hospitals:

University of Gothenburg and Sahlgrenska University Hospital; Karolinska Institutet and Karolinska University Hospital; Linköping University and Linköping University Hospital; Lund University and Skåne University Hospital; Umeå University and University Hospital of Umeå; Uppsala University and Uppsala University Hospital.

## Scientific Advisory Board

Marike Boezen, University of Groningen, Groningen, NL; Göran Berglund, Skåne University Hospital and Lund University, Malmö, SE; Robert Clarke, University of Oxford, UK; Marc Dewey, Radiology Institute at Charité University of Medicine, Berlin, GE; Ulf De Faire, Karolinska Institutet, Solna, SE; KayTee Khaw, University of Cambridge, UK; Juhani Knuuti, Turku PET Centre, Turku University Hospital, Turku, FI; Claes-Göran Löfdahl, Skåne University Hospital and Lund University, Lund, SE; Eva Prescott,University of Copenhagen and Bispebjerg University Hospital, Copenhagen, DK; Anders Waldenström,Umeå University, Umeå, SE

## Key persons involved in early discussions

Göran Berglund, Göran Bergström, Björn Fagerberg, Ulf de Faire, Jan Engvall, Bo Hedblad, Christer

Janson, Lars Johansson, Lars Lind, Claes-Göran Löfdahl, Fredrik Nyström, Anders Persson, Annika

Rosengren, Stefan Söderberg, Anders Waldenström, Hans Wedel

## Supportive Organization & Coordinating site

The Swedish Heart and Lung Foundation [2019-0012] is the main funder of SCAPIS. In addition to

providing financial support, the Swedish Heart and Lung Foundation has facilitated this national

collaborative project by assisting in arranging study meetings, providing travel support and meeting venues,

and sharing their expertise in communicating science.

### Swedish Heart and Lung Foundation

Kristina Sparreljung (secretary general), Staffan Josephsson (former secretary general), Mira Ernkvist, Bim

Boberg, Amanda Skog Andreasson, Ebba Bergman, Anna Fredholm, Louise Fornander, Susanne Klofsten,

Christa Larsvall, Anna Sjöström, Sofia Swedenborg, Joanna Tingström, Caroline Waldenström Sylvén,

Sofia Wirsén, Jan Nilsson, Anders Waldenström

### Study operations

#### Operational Management Team

Eva Karin Anderberg, Anna Andreasson, Göran Bergström, Anna Beskow, Bim Boberg, Charlotta Elfström, Mira Ernkvist, Peter Hedman, Carl Johan Östgren

#### SCAPIS Office

Eva Karin Anderberg (coordinator), Kristina Levan, Åsa Odhagen Rosvall, Charlotte Benninge

#### National Project Managers

National Project Coordinator: Charlotta Elfström

Project Managers: Sven Anders Benjegård, Ebba Bergman, Martin Brandhagen, Sven Burman, Olivia

Claesson, Louise Fornander, Anna Frick, Urban Gustafsson, Emma Larsson, Maria Matson Dzebo, Tina

Noord, Klara Thorsson

#### Data Management

IT Project Lead: Tina Noord (main coordinator), Anita Adolfsson, Anna Andreasson

Product Owner and Product Specialist: Anna Frick, Christian Johansen, Emma Larsson, Theodor Lewén

IT Development Team: Solution Architects: Christian Johansen, Tomas Snäckerström, Andreas Wallén; *System Developers*: Simon Dirnberger, Johan Fredin, Albin Willman, Max Jourdanis

Study Data Management: Niklas Svensson

Image Data Storage: Emanuel Hillberg, Nasser Hosseini, Åke Marjamäki, Tomas Moberg

Accelerometry Data Management: Örjan Ekblom, Elin Ekblom-Bak

Local IT Support: *Gothenburg*: Anders Broman, Johan Fors, Roger Lampa; *Linköping:* Tomas Annerholm;

*Malmö*: Pawel Gagol; *Umeå*: Gunnar Jonsson, Fredrik Lejon, Mattias Wennberg; *Uppsala*: Jörgen Anell,

Martin Karlsson, José Conde Herrera

#### Head Project Statistician

Erik Lampa

#### Publication Support

Ebba Bergman, Louise Fornander, Sofia Swedenborg, Ruby Rahman, Rebecca Josefson

#### Senior Scientific Editor

Rosie Perkins

## Local Project Lead, Coordinators & Local Steering Committee

### University of Gothenburg

*Principal Investigators*: Göran Bergström (PI), Annika Rosengren (co-PI); *Local Coordinator*: Caroline

Schmidt; *Local Steering Committee Members*: Oskar Angerås, John Brandberg, Jan Hedner, Kjell Torén

### Linköping University

*Principal Investigators*: Carl Johan Östgren (PI), Jan Engvall (co-PI); *Local Coordinators*: Charlotte Brage,

Elisabeth Logander; *Local Steering Committee Members*: Preben Bendtsen, Per Dannetun, Kjell Jansson,

Magnus Janzon, Ditte Pehrsson-Lindell, Anders Persson, Mats Ulfendahl

### Lund University

*Principal Investigators*: Gunnar Engström (PI), Margaretha Persson (co-PI); *Local Coordinator*: Margaretha

Persson; *Local Steering Committee Members*: Lars Bååth, Joyce Carlsson, Arne Egesten, Olle Ekberg,

David Erlinge, Isabel Goncalves, Anders Gottsäter, Bo Hedblad, Martin Magnusson, Olle Melander, Peter

Nilsson, Eeva Piitulainen, Per Wollmer, Gerd Östling

### Karolinska Institute

*Principal Investigators*: Tomas Jernberg (PI), Magnus Sköld (co-PI); *Local Coordinators*: Liselotte Persson,

Sven Burman, Erica Ottenblad, Teresa Sandvall, Cecilia Ström; Local *Steering Committee Members*:

Kenneth Caidahl, Kerstin Cederlund, Maria Englund, Mats Eriksson, Maria Eriksson, Per Eriksson, Maria

Mannila

### Umeå University

*Principal Investigators*: Anders Blomberg (PI), Stefan Söderberg (co-PI); *Local Coordinator*: Kristin Ahlm,

Anna Ramnemark; *Local Steering Committee Members*: Diana Berggren, Kjell Burman, Karl Gustav

Forsberg, Magnus Hedström, Marlene Landström, Per Lindqvist, Cecilia Mattsson, Anna Ramnemark,

Anette Sandström, Marie Strand, Jenny Åkerblom

### Uppsala University

*Principal Investigators*: Johan Sundström (PI), Lars Lind (co-PI); *Local Coordinators*: Maria Storgärds;

*Local Steering Committee Members*: Håkan Ahlström, Olov Duvernoy, Tove Fall, Emil Hagström, Stefan

James, Christer Janson, Sune Larsson, Eva Lindberg, Andrei Malinovschi, Jonas Oldgren

## Scientific Working Groups

Lars Lind (Chair of the Biomarker Group), Kjell Torén (Chair of the Lung and Pulmonary Group), Carl

Johan Östgren (Chair of the Metabolism Group), Gunnar Engström (Chair of the Cohort Group), Jan

Engvall (Chair of the Image Analysis Group)

## Biobank Advisory Resources

Anna Beskow, Sonja Eaker Fält, Margaretha Persson, Gunnel Tybring

## Quality Managers

*Ultrasonography*: Caroline Schmidt, Gerd Östling; *Computed Tomography (CT):* John Brandberg, Marit Johannesson, Helén Milde; *Coronary CT Angiography*: Lilian Henriksson; *Pulmonary Function and Vital Signs*: Annika Johansson, Cecilia Kennbäck

## Study execution

### Study Physicians

*Gothenburg*: Noraldeen Al-Dury, Anna Björk, Matilda Du Rietz, Hanna Eriksson, Magnus Hallor, Mattias

Hallsten, Karin Hedman, Anna Jeppson, Josefina Robertsson, Sara Roos, Jessica Sjölund, Linda

Thorvaldsson, Johan Thurell, Adam Zachrisson; *Linköping*: Eva Swahn (national convenor); *Malmö*:

Erasmus Bachus, Klas Gränsbo, Viktor Hamrefors; *Stockholm:* Maria Mannila, Gundars Rasmanis, Izabella

Zarea-Ganji; *Umeå*: Shariar Fezi Razi, Anja Isaksson, Lars Nilsson, Anette Sandström, Linn Skoglund;

*Uppsala*: Johan Forsblad, Emil Hagström, Martin Sandelin

### SCAPIS Site-Responsible Nurse/Team Lead

*Gothenburg*: Catherine Åhlund; *Linköping*: Elisabeth Logander; *Malmö*: Cecilia Kennbäck; *Stockholm*:

Caroline Bäck, Pia Löf, Emma Stahre; *Umeå*: Kristin Ahlm; *Uppsala*: Aregash Tesfaldet

### SCAPIS Site-Responsible Radiology Nurse

*Gothenburg*: Marit Johannesson, Helen Milde; *Linköping*: Lilian Henriksson; *Malmö*: Elisabeth Andersson;

*Stockholm*: Aziza Adem; *Umeå*: Maria Lundbäck, David Wahllöf; *Uppsala*: Monica Segelsjö

### Radiologists and cardiologists – Angiography

(in numerical order, starting with highest number of examinations/radiologist.)

Agneta Flinck (Gothenburg), Olov Duvernoy (Uppsala), Anders Hauggaard (Linköping), Raquel Themudo

(Stockholm), Tanja Kero (Uppsala), Ellen Ostenfeld (Malmö), Lisa Ander Olsson (Malmö), Kerstin

Cederlund (Stockholm), Catharina Adlercreutz (Malmö), Hans Lindgren (Umeå), Karen Sörensen (Umeå),

Susann Skoog (Linköping), Erika Fagman (Gothenburg), Maria Kjellin (Linköping), Katharina Brehmer

(Stockholm), Franciska Wikner (Umeå), Isabel Goncalves (Malmö), Hanna Markstad (Malmö), Anders

Björkholm (Linköping), Caroline Berntsson (Gothenburg), Marcus Gjerde (Linköping), Elin Bacsovics

Brolin (Stockholm), Louise Norlén (Stockholm), Johan Blomma (Linköping), Gunnar Wiklund (Linköping),

Lisbeth Denbratt (Gothenburg), Adrian Pistea (Malmö), Kerstin Cederlund (Linköping), Artur Tomson

(Stockholm), Margareta Klein (Stockholm), Gusten Nyberg (Linköping), Tomasz Baron (Uppsala), Ylva

Gårdinger (Malmö), Viktor Hamrefors (Malmö), Sofia Olai (Linköping), Ming Chen (Stockholm)

### Radiologists – Emphysema

(in numerical order, starting with highest number of examinations/radiologist.)

Johan Thurén (Uppsala), Marianne Boijsen (Gothenburg), Ulf Molin (Uppsala), Catharina Adlercreutz

(Malmö), Anders Hauggaard (Linköping), Sören Strandberg (Stockholm), Lars Bååth (Malmö), Jenny

Vikgren (Gothenburg), Kerstin Cederlund (Stockholm), Hans Lindgren (Umeå), Susann Skoog (Linköping),

Lisa Ander Olsson (Malmö), Karen Sörensen (Umeå), Maria Kjellin (Linköping), Franciska Wikner

(Umeå), Egon Wallier (Stockholm), Katharina Brehmer (Stockholm), Anders Björkholm (Linköping), Johan

Blomma (Linköping), Hanna Markstad (Malmö), Gunnar Wiklund (Linköping), Bengt Gottfridsson

(Gothenburg), Gusten Nyberg (Linköping), Andreas Malmqvist (Stockholm), Kerstin Cederlund

(Linköping), Dariusz Slusarczyk (Malmö), Raquel Themudo (Stockholm), Bertil Larsson (Uppsala), Anna

Kahn (Malmö), Rauni Rossi Norrlund (Gothenburg), Olov Duvernoy (Uppsala), Sofia Olai (Linköping),

Charlotta Lidbjörk (Linköping), Åse Johnsson (Gothenburg), Tomas Hansen (Uppsala)

### Other Personnel Categories of Importance

Biomedical analysts, radiology nurses, clinical and assistant nurses at the six sites. Other persons involved in the governance, operation and execution of the project.

### SCAPIS Site-Responsible Organization

*Gothenburg:* Gothia Forum; *Linköping*: Department of Clinical Physiology, Linköping University Hospital;

*Malmö*: Clinical Research Unit, Department of Medicine, Skåne University Hospital, Malmö; *Stockholm*:

Karolinska Trial Alliance (KTA); *Umeå*: Clinical Research Center, Region; *Uppsala*: Uppsala Clinical Research Center/Uppsala Biobank, Uppsala University

Legends

Figure S1. The cumulative distribution of FEV_1_/FVC by smoking status. The vertical line represents the GOLD cut off for CAL, i.e., 0.7.

Figure S2. Complementary cumulative distribution of emphysema score for three smoking status groups. The lines show the percentage of the smoking status groups with an emphysema score higher than corresponding position on x-axis.

Figure S3. Venn diagram illustrating the co-occurrence of CAL, emphysema and impaired DLco in ever-smokers (n =3,439) (Panel A) and never-smokers (n =1,706) (Panel B), respectively. Note that the circles are proportional to prevalence of CAL, emphysema and impaired DLco within, but not between, smoking status groups.

Figure S4. Unadjusted prevalence ratios for respiratory symptoms (breathlessness (upper panel), chronic bronchitis (middle panel) and wheeze (lower panel)) by CAL and smoking status (left panel), by emphysema and smoking status (middle panel) and by impaired DLCO and smoking status (right panel). Red colour intensity is proportional to magnitude of prevalence ratio.

Figure S5. Adjusted prevalence ratios for respiratory symptoms (breathlessness (upper panel), chronic bronchitis (middle panel) and wheeze (lower panel)) by CAL and smoking status (left panel), by emphysema and smoking status (middle panel) and by impaired DLCO and smoking status (right panel) when excluding individuals with self-reported asthma, diagnosed before 40 years of age. Red colour intensity is proportional to magnitude of prevalence ratio.

| Table S1. Lung physiology, airway symptoms and comorbidities by CAL and smoking status, categorized as never/ever-smokers. | | | | |
| --- | --- | --- | --- | --- |
|  | **No CAL** | | **CAL** | |
|  | **Never-smoker**  N = 13,755 | **Ever-smoker**  N = 12,473 | **Never-smoker**  N = 803 | **Ever-smoker**  N = 1,715 |
| Emphysema | 261 (1.9%) | 839 (6.8%) | 40 (5.0%) | 489 (29%) |
| DLco (% predicted) | 99 (91, 108) | 96 (87, 106) | 101 (91, 110) | 89 (77, 101) |
| DLco < LLN | 705 (5.3%) | 1,225 (10%) | 51 (6.5%) | 486 (29%) |
| FEV_1_ (% predicted) | 100 (92, 108) | 98 (90, 107) | 87 (78, 96) | 82 (72, 91) |
| FEV_1_ < LLN | 739 (5.4%) | 855 (6.9%) | 252 (31%) | 767 (45%) |
| Breathlessness | 440 (3.2%) | 591 (4.8%) | 40 (5.0%) | 211 (12%) |
| Chronic bronchitis | 475 (3.5%) | 637 (5.2%) | 56 (7.1%) | 229 (14%) |
| Wheeze | 561 (4.1%) | 1,008 (8.2%) | 110 (14%) | 407 (24%) |
| Asthma | 651 (4.8%) | 532 (4.4%) | 110 (14%) | 168 (10%) |
| Ischemic heart disease | 238 (1.7%) | 356 (2.9%) | 21 (2.6%) | 91 (5.3%) |
| CACS > 100 | 2,019 (15%) | 2,730 (23%) | 135 (17%) | 554 (34%) |
| Diabetes mellitus | 845 (6.1%) | 1,048 (8.4%) | 45 (5.6%) | 169 (9.9%) |
| hsCRP >3 (mg/l) | 1,908 (14%) | 2,263 (18%) | 110 (14%) | 403 (24%) |
| Pack years of cigarette smoking |  | 12 (5.0, 22) |  | 22 (10, 34) |

Data are given as median (IQR) or N (%)

| Table S2. Lung physiology, airway symptoms and comorbidities by emphysema and smoking status, categorized as never/ever-smokers | | | | |
| --- | --- | --- | --- | --- |
|  | No emphysema | | Emphysema | |
|  | **Never-smoker**,  N = 14,068 | **Ever-smoker**,  N = 12,645 | **Never-smoker**,  N = 301 | **Ever-smoker**,  N = 1,328 |
| CAL | 755 (5.4%) | 1,189 (9.4%) | 40 (13%) | 489 (37%) |
| DLco (% predicted) | 99 (91, 108) | 96 (87, 106) | 97 (87, 106) | 85 (73, 96) |
| DLco < LLN | 713 (5.2%) | 1,198 (9.8%) | 29 (9.9%) | 473 (37%) |
| FEV_1_ (% predicted) | 99 (91, 107) | 98 (89, 106) | 97 (88, 106) | 90 (79, 100) |
| FEV_1_ < LLN | 936 (6.7%) | 1,221 (9.7%) | 34 (11%) | 360 (27%) |
| Breathlessness | 459 (3.3%) | 640 (5.1%) | 8 (2.7%) | 136 (10%) |
| Chronic bronchitis | 511 (3.7%) | 701 (5.7%) | 10 (3.4%) | 150 (12%) |
| Wheeze | 650 (4.7%) | 1,159 (9.4%) | 15 (5.0%) | 219 (17%) |
| Asthma | 729 (5.3%) | 620 (5.0%) | 19 (6.4%) | 69 (5.4%) |
| Ischemic heart disease | 249 (1.8%) | 374 (3.0%) | 9 (3.0%) | 65 (4.9%) |
| CACS > 100 | 2,097 (15%) | 2,833 (23%) | 55 (19%) | 446 (35%) |
| Diabetes mellitus | 867 (6.2%) | 1,077 (8.5%) | 13 (4.3%) | 119 (9.0%) |
| hsCRP >3 (mg/l) | 1,949 (14%) | 2,280 (18%) | 46 (15%) | 336 (25%) |
| Pack years of cigarette smoking |  | 12 (4.8, 21) |  | 25 (15, 36) |

Data are given as median (IQR) or N (%)

| Table S3. Lung physiology, airway symptoms and comorbidities by DLco and smoking status, categorized as never/ever-smokers | | | | |
| --- | --- | --- | --- | --- |
|  | **Normal DLco** | | **Impaired DLco** | |
|  | **Never-smoker**  N = 13,380 | **Ever-smoker**  N = 12,052 | **Never-smoker**  N = 756 | **Ever-smoker**  N = 1,711 |
| CAL | 737 (5.5%) | 1,184 (9.8%) | 51 (6.7%) | 486 (28%) |
| Emphysema | 264 (2.0%) | 809 (6.8%) | 29 (3.9%) | 473 (28%) |
| FEV_1_ (% predicted) | 100 (92, 108) | 98 (90, 107) | 90 (81, 99) | 86 (76, 95) |
| FEV_1_ < LLN | 765 (5.7%) | 964 (8.0%) | 183 (24%) | 588 (34%) |
| Breathlessness | 398 (3.0%) | 538 (4.5%) | 62 (8.3%) | 232 (14%) |
| Chronic bronchitis | 489 (3.7%) | 643 (5.5%) | 31 (4.2%) | 186 (11%) |
| Wheeze | 597 (4.5%) | 1,034 (8.7%) | 56 (7.5%) | 320 (19%) |
| Asthma | 718 (5.5%) | 601 (5.1%) | 25 (3.4%) | 81 (4.9%) |
| Ischemic heart disease | 212 (1.6%) | 310 (2.6%) | 32 (4.2%) | 111 (6.5%) |
| CACS > 100 | 1,977 (15%) | 2,630 (23%) | 119 (17%) | 532 (33%) |
| Diabetes mellitus | 783 (5.9%) | 960 (8.0%) | 75 (9.9%) | 216 (13%) |
| hsCRP >3 (mg/l) | 1,754 (13%) | 2,077 (17%) | 184 (24%) | 504 (30%) |
| Pack years of cigarette smoking |  | 11 (4.8, 21) |  | 22 (12, 34) |

Data are given as median (IQR) or N (%)

Table S4. Site specific prevalence of CAL, Emphysema and impaired DLco with 95% confidence interval.

| Site | CAL | Emphysema | Impaired DLco |
| --- | --- | --- | --- |
| Gothenburg (n = 6071) | 9.0% (8.3, 9.7) | 6.0% (5.4, 6.6) | 9.4% (8.7, 10) |
| Linköping (n = 4822) | 8.8% (8.0, 9.6) | 6.5% (5.8, 7.2) | 5.2% (4.6, 5.9) |
| Malmö (n = 5957) | 8.2% (7.5,8.9) | 6.6% (5.9, 7.2) | 15% (14, 16) |
| Stockholm (n = 4917) | 12% (11, 13) | 6.7% (6.0, 7.4) | 4.5% (4.0, 5.2) |
| Umeå (n = 2349) | 6.8% (5.8, 7.9) | 6.9% (5.9, 8.0) | 10% (9.1, 12) |
| Uppsala (n = 4630) | 6.9% (6.2, 7.7) | 2.1% (1.7, 2.5) | 7.8% (7.0, 8.6) |

| Table S5. Airway symptoms and comorbidities by CAL and smoking status for males | | | | | | |
| --- | --- | --- | --- | --- | --- | --- |
|  | **No CAL** | | | **CAL** | | |
|  | **Never-smoker**  N=6,905 | **Ex-smoker**  N = 4,202 | **Current smoker**  N = 1,400 | **Never-smoker**  N = 530 | **Ex-smoker**  N = 512 | **Current smoker**  N = 384 |
| Emphysema | 139 (2.0%) | 246 (5.9%) | 201 (15%) | 26 (4.9%) | 101 (20%) | 158 (42%) |
| DLco (% predicted) | 99 (91, 108) | 98 (89, 107) | 92 (82, 101) | 101 (92, 109) | 96 (85, 107) | 81 (70, 94) |
| DLco < LLN | 311 (4.6%) | 301 (7.4%) | 241 (18%) | 33 (6.3%) | 66 (13%) | 168 (45%) |
| FEV_1_ (% predicted) | 100 (92, 108) | 99 (91, 107) | 96 (88, 104) | 87 (77, 96) | 84 (77, 92) | 79 (69, 91) |
| FEV_1_ < LLN | 331 (4.8%) | 233 (5.5%) | 135 (9.6%) | 162 (31%) | 172 (34%) | 196 (51%) |
| Breathlessness | 115 (1.7%) | 132 (3.2%) | 57 (4.1%) | 22 (4.2%) | 34 (6.6%) | 49 (13%) |
| Chronic bronchitis | 240 (3.5%) | 204 (5.0%) | 132 (9.8%) | 34 (6.5%) | 61 (12%) | 74 (20%) |
| Wheeze | 258 (3.8%) | 268 (6.5%) | 199 (15%) | 69 (13%) | 81 (16%) | 113 (31%) |
| Asthma | 292 (4.3%) | 152 (3.7%) | 30 (2.2%) | 68 (13%) | 56 (11%) | 29 (7.8%) |
| Ischemic heart disease | 182 (2.6%) | 196 (4.7%) | 51 (3.6%) | 20 (3.8%) | 35 (6.8%) | 28 (7.3%) |
| CACS > 100 | 1,568 (24%) | 1,333 (34%) | 470 (35%) | 112 (22%) | 199 (41%) | 163 (46%) |
| Diabetes mellitus | 522 (7.6%) | 475 (11%) | 162 (12%) | 38 (7.2%) | 53 (10%) | 53 (14%) |
| hsCRP >3 (mg/l) | 833 (12%) | 672 (16%) | 301 (22%) | 68 (13%) | 100 (20%) | 98 (26%) |
| Pack years of cigarette smoking |  | 10 (4.5, 20) | 20 (10, 33) |  | 14 (5.7, 27) | 33 (19, 41) |

Data are given as median (IQR) or N (%)

| Table S6. Airway symptoms and comorbidities by CAL and smoking status for females | | | | | | |
| --- | --- | --- | --- | --- | --- | --- |
|  | **No CAL** | | | **CAL** | | |
|  | **Never-smoker**  N=6,850 | **Ex-smoker**  N = 5,299 | **Current smoker**  N = 1,572 | **Never-smoker**  N = 273 | **Ex-smoker**  N = 487 | **Current smoker**  N=332 |
| Emphysema | 122 (1.8%) | 225 (4.3%) | 167 (11%) | 14 (5.2%) | 99 (21%) | 131 (41%) |
| DLco (% predicted) | 99 (91, 108) | 98 (89, 107) | 91 (82, 101) | 101 (90, 110) | 92 (82, 102) | 81 (70, 91) |
| DLco < LLN | 394 (5.9%) | 361 (7.0%) | 322 (21%) | 18 (6.7%) | 97 (20%) | 155 (48%) |
| FEV_1_ (% predicted) | 100 (92, 107) | 99 (91, 108) | 96 (88, 106) | 87 (79, 94) | 83 (74, 92) | 78 (67, 87) |
| FEV_1_ < LLN | 408 (6.0%) | 311 (5.9%) | 176 (11%) | 90 (33%) | 213 (44%) | 186 (56%) |
| Breathlessness | 325 (4.8%) | 298 (5.6%) | 104 (6.7%) | 18 (6.6%) | 70 (14%) | 58 (18%) |
| Chronic bronchitis | 235 (3.5%) | 204 (3.9%) | 97 (6.4%) | 22 (8.3%) | 37 (7.8%) | 57 (18%) |
| Wheeze | 303 (4.5%) | 305 (5.8%) | 236 (15%) | 41 (15%) | 85 (18%) | 128 (40%) |
| Asthma | 359 (5.3%) | 277 (5.3%) | 73 (4.8%) | 42 (16%) | 57 (12%) | 26 (8.3%) |
| Ischemic heart disease | 56 (0.8%) | 83 (1.6%) | 26 (1.7%) | 1 (0.4%) | 19 (3.9%) | 9 (2.7%) |
| CACS > 100 | 451 (6.7%) | 667 (13%) | 260 (17%) | 23 (8.6%) | 99 (21%) | 93 (29%) |
| Diabetes mellitus | 323 (4.7%) | 301 (5.7%) | 110 (7.0%) | 7 (2.6%) | 30 (6.2%) | 33 (9.9%) |
| hsCRP >3 (mg/l) | 1,075 (16%) | 968 (18%) | 322 (21%) | 42 (15%) | 112 (23%) | 93 (28%) |
| Pack years of cigarette smoking |  | 9.0 (4.0, 17) | 20 (11, 30) |  | 16 (6.5, 26) | 30 (20, 38) |

Data are given as median (IQR) or N (%)

| Tabell S7. Airway symptoms and comorbidities by emphysema and smoking status for males. | | | | | | |
| --- | --- | --- | --- | --- | --- | --- |
|  | **No emphysema** | | | **Emphysema** | | |
|  | **Never-smoker**  N =7,200 | **Ex-smoker**  N = 4,299 | **Current smoker**  N = 1,397 | **Never-smoker**  N = 165 | **Ex-smoker**  N = 347 | **Current smoker**  N = 359 |
| CAL | 500 (6.9%) | 405 (9.4%) | 214 (15%) | 26 (16%) | 101 (29%) | 158 (44%) |
| DLco (% predicted) | 99 (91, 108) | 98 (89, 107) | 92 (82, 101) | 98 (87, 105) | 93 (82, 104) | 79 (69, 90) |
| DLco < LLN | 318 (4.5%) | 296 (7.1%) | 226 (17%) | 18 (11%) | 65 (19%) | 174 (50%) |
| FEV_1_ (% predicted) | 99 (91, 107) | 98 (90, 106) | 95 (85, 104) | 98 (88, 106) | 93 (83, 103) | 88 (76, 98) |
| FEV_1_ < LLN | 465 (6.5%) | 342 (8.0%) | 210 (15%) | 21 (13%) | 57 (16%) | 114 (32%) |
| Breathlessness | 132 (1.8%) | 139 (3.2%) | 64 (4.6%) | 3 (1.8%) | 24 (6.9%) | 39 (11%) |
| Chronic bronchitis | 265 (3.7%) | 221 (5.3%) | 142 (11%) | 6 (3.7%) | 40 (12%) | 60 (18%) |
| Wheeze | 314 (4.4%) | 303 (7.2%) | 223 (17%) | 10 (6.1%) | 40 (12%) | 82 (24%) |
| Asthma | 349 (4.9%) | 188 (4.5%) | 46 (3.4%) | 10 (6.2%) | 18 (5.4%) | 11 (3.2%) |
| Ischemic heart disease | 195 (2.7%) | 206 (4.8%) | 56 (4.0%) | 6 (3.6%) | 23 (6.6%) | 20 (5.6%) |
| CACS > 100 | 1,634 (23%) | 1,387 (34%) | 485 (36%) | 44 (27%) | 142 (43%) | 148 (42%) |
| Diabetes mellitus | 542 (7.5%) | 478 (11%) | 173 (12%) | 11 (6.7%) | 40 (12%) | 41 (11%) |
| hsCRP >3 (mg/l) | 870 (12%) | 679 (16%) | 286 (21%) | 23 (14%) | 82 (24%) | 103 (29%) |
| Pack years of cigarette smoking |  | 10 (4.5, 20) | 20 (10, 34) |  | 21 (12, 32) | 32 (19, 40) |

Data are given as median (IQR) or N (%)

| Tabell S8. Airway symptoms and comorbidities by emphysema and smoking status for females. | | | | | | |
| --- | --- | --- | --- | --- | --- | --- |
|  | **No emphysema** | | | **Emphysema** | | |
|  | **Never-smoker**  N = 6,868 | **Ex-smoker**  N = 5,384 | **Current smoker**  N = 1,565 | **Never-smoker**  N = 136 | **Ex-smoker**  N = 324 | **Current smoker**  N = 298 |
| CAL | 255 (3.7%) | 378 (7.0%) | 192 (12%) | 14 (10%) | 99 (31%) | 131 (44%) |
| DLco (% predicted) | 100 (91, 109) | 98 (89, 107) | 91 (82, 10) | 97 (88, 108) | 90 (78, 99) | 80 (68, 89) |
| DLco < LLN | 395 (5.9%) | 359 (6.8%) | 317 (21%) | 11 (8.3%) | 90 (28%) | 144 (50%) |
| FEV_1_ (% predicted) | 99 (91, 107) | 99 (90, 107) | 95 (85, 105) | 96 (88, 105) | 93 (80, 103) | 87 (75, 97) |
| FEV_1_ < LLN | 471 (6.9%) | 425 (7.9%) | 244 (16%) | 13 (9.6%) | 84 (26%) | 105 (35%) |
| Breathlessness | 327 (4.8%) | 321 (6.0%) | 116 (7.5%) | 5 (3.7%) | 36 (11%) | 37 (13%) |
| Chronic bronchitis | 246 (3.7%) | 221 (4.2%) | 117 (7.7%) | 4 (3.0%) | 16 (5.1%) | 34 (12%) |
| Wheeze | 336 (5.0%) | 352 (6.6%) | 281 (18%) | 5 (3.7%) | 28 (8.8%) | 69 (24%) |
| Asthma | 380 (5.6%) | 305 (5.8%) | 81 (5.4%) | 9 (6.6%) | 24 (7.5%) | 16 (5.6%) |
| Ischemic heart disease | 54 (0.8%) | 86 (1.6%) | 26 (1.7%) | 3 (2.2%) | 15 (4.6%) | 7 (2.3%) |
| CACS > 100 | 463 (6.8%) | 698 (13%) | 263 (17%) | 11 (8.2%) | 68 (22%) | 88 (30%) |
| Diabetes mellitus | 325 (4.7%) | 307 (5.7%) | 119 (7.6%) | 2 (1.5%) | 19 (5.9%) | 19 (6.4%) |
| hsCRP >3 (mg/l) | 1,079 (16%) | 988 (18%) | 327 (21%) | 23 (17%) | 73 (23%) | 78 (26%) |
| Pack years of cigarette smoking |  | 9.0 (4.0, 17) | 20 (11, 30) |  | 21 (12, 31) | 29 (20, 37) |

Data are given as median (IQR) or N (%)

| Tabell S9. Airway symptoms and comorbidities by DLco and smoking status for males. | | | | | | |
| --- | --- | --- | --- | --- | --- | --- |
|  | **No impaired DLco** | | | **Impaired DLco** | | |
|  | **Never-smoker**  N =6,898 | **Ex-smoker**  N =4,208 | **Current smoker**  N = 1,296 | **Never-smoker**  N = 344 | **Ex-smoker**  N = 367 | **Current smoker**  N = 409 |
| CAL | 488 (7.1%) | 436 (10%) | 205 (16%) | 33 (9.6%) | 66 (18%) | 168 (41%) |
| Emphysema | 143 (2.1%) | 269 (6.5%) | 171 (13%) | 18 (5.4%) | 65 (18%) | 174 (44%) |
| FEV_1_ (% predicted) | 100 (91, 107) | 98 (90, 107) | 96 (87, 104) | 89 (79, 97) | 87 (77, 97) | 84 (75, 94) |
| FEV_1_ < LLN | 384 (5.6%) | 286 (6.8%) | 158 (12%) | 94 (27%) | 104 (28%) | 158 (39%) |
| Breathlessness | 113 (1.6%) | 116 (2.8%) | 50 (3.9%) | 21 (6.2%) | 45 (12%) | 49 (12%) |
| Chronic bronchitis | 259 (3.8%) | 228 (5.5%) | 121 (9.7%) | 14 (4.1%) | 30 (8.4%) | 72 (18%) |
| Wheeze | 298 (4.4%) | 301 (7.3%) | 199 (16%) | 23 (6.8%) | 41 (12%) | 91 (23%) |
| Self-reported asthma | 345 (5.1%) | 185 (4.5%) | 44 (3.5%) | 7 (2.1%) | 16 (4.5%) | 13 (3.3%) |
| Ischemic heart disease | 168 (2.4%) | 174 (4.1%) | 50 (3.9%) | 25 (7.3%) | 40 (11%) | 26 (6.4%) |
| CACS > 100 | 1,546 (23%) | 1,343 (34%) | 421 (34%) | 89 (28%) | 140 (42%) | 181 (47%) |
| Diabetes mellitus | 494 (7.2%) | 444 (11%) | 147 (11%) | 48 (14%) | 63 (17%) | 60 (15%) |
| hsCRP >3 (mg/l) | 794 (12%) | 648 (15%) | 240 (19%) | 75 (22%) | 102 (28%) | 137 (34%) |
| Pack years of cigarette smoking |  | 10 (4.5, 20) | 20 (9.9, 33) |  | 16 (6.8, 29) | 32 (18, 40) |

Data are given as median (IQR) or N (%)

| Tabell S10. Airway symptoms and comorbidities by DLco and smoking status for females. | | | | | | |
| --- | --- | --- | --- | --- | --- | --- |
|  | **No impaired DLco** | | | **DLco** | | |
|  | **Never-smoker**  N = 6,868 | **Ex-smoker**  N = 5,384 | **Current smoker**  N = 1,565 | **Never-smoker**  N = 136 | **Ex-smoker**  N = 324 | **Current smoker**  N = 298 |
| CAL | 249 (3.8%) | 378 (7.3%) | 165 (12%) | 18 (4.4%) | 97 (21%) | 155 (32%) |
| Emphysema | 121 (1.9%) | 227 (4.4%) | 142 (11%) | 11 (2.7%) | 90 (20%) | 144 (31%) |
| FEV_1_ (% predicted) | 100 (92, 108) | 99 (91, 107) | 97 (88, 106) | 91 (83, 100) | 88 (78, 96) | 84 (74, 95) |
| FEV_1_ < LLN | 381 (5.9%) | 366 (7.1%) | 154 (11%) | 89 (22%) | 138 (30%) | 188 (39%) |
| Breathlessness | 285 (4.4%) | 285 (5.5%) | 87 (6.5%) | 41 (10%) | 69 (15%) | 69 (15%) |
| Chronic bronchitis | 230 (3.6%) | 206 (4.0%) | 88 (6.7%) | 17 (4.2%) | 28 (6.3%) | 56 (12%) |
| Wheeze | 299 (4.7%) | 324 (6.3%) | 210 (16%) | 33 (8.2%) | 52 (12%) | 136 (30%) |
| Self-reported asthma | 373 (5.9%) | 303 (6.0%) | 69 (5.2%) | 18 (4.5%) | 25 (5.6%) | 27 (5.9%) |
| Ischemic heart disease | 44 (0.7%) | 76 (1.5%) | 10 (0.7%) | 7 (1.7%) | 22 (4.8%) | 23 (4.8%) |
| CACS > 100 | 431 (6.8%) | 649 (13%) | 217 (16%) | 30 (7.6%) | 96 (22%) | 115 (26%) |
| Diabetes mellitus | 289 (4.5%) | 279 (5.4%) | 90 (6.6%) | 27 (6.6%) | 46 (10%) | 47 (9.9%) |
| hsCRP >3 (mg/l) | 960 (15%) | 936 (18%) | 253 (19%) | 109 (26%) | 116 (25%) | 149 (31%) |
| Pack years of cigarette smoking |  | 9.0 (4.0, 17) | 19 (11, 29) |  | 16 (7.1, 26) | 28 (20, 36) |

| Data are given as median (IQR) or N (%) |
| --- |
|  |
|  |

Table S11a. Interaction effects between CAL and current smoking on prevalence of breathlessness. PR – prevalence ratio

|  | **No CAL** | | **CAL** | |  |
| --- | --- | --- | --- | --- | --- |
|  | N breathlessness /no breathlessness | PR (95% CI) | N breathlessness/no breathlessness | PR (95% CI) | PR (95% CI) for CAL within smoking strata |
| **Never-smokers** | 440/13,276 | 1 | 40/759 | 1.56 (1.14, 2.14) | 1.56 (1.14, 2.14) |
| **Current smokers** | 161/2780 | 1.71(1.43, 2.04) | 107/598 | 4.73 (3.88, 5.76) | 2.78 (2.17, 3.54) |

Measure of interaction on multiplicative scale: 1.77; 95% CI: [1.18, 2.66]; p = 0.005

Prevalence ratios (PR) are calculated from unadjusted models.

Table S11b. Interaction effects between CAL and current smoking on prevalence of chronic bronchitis. PR – prevalence ratio

|  | **No CAL** | | **CAL** | |  |
| --- | --- | --- | --- | --- | --- |
|  | N chronic bronchitis/no chronic bronchitis | PR (95% CI) | N chronic bronchitis /no chronic bronchitis | PR (95% CI) | PR (95% CI) for CAL within smoking strata |
| **Never-smokers** | 475/13,067 | 1 | 56/733 | 2.02 (1.55, 2.64) | 2.02 (1.55, 2.64) |
| **Current smokers** | 229/2,630 | 2.28 (1.96, 2.66) | 131/547 | 5.51 (4.61, 6.58) | 2.41 (1.95, 2.99) |

Measure of interaction on multiplicative scale: 1.19; 95% CI: [0.84, 1.69]; p = 0.33

Prevalence ratios (PR) are calculated from unadjusted models.

Table S11c. Interaction effects between CAL and current smoking on prevalence of wheeze. PR – prevalence ratio

|  | **No CAL** | | **CAL** | |  |
| --- | --- | --- | --- | --- | --- |
|  | N wheeze/no wheeze | PR (95% CI) | N wheeze/no wheeze | PR (95% CI) | PR (95% CI) for CAL within smoking strata |
| **Never-smokers** | 561/13,004 | 1 | 110/684 | 3.35 (2.77, 4.06) | 3.35 (2.77, 4.06) |
| **Current smokers** | 435/2439 | 3.66 (3.25, 4.12) | 241/441 | 8.54 (7.50, 9.73) | 2.33 (1.99, 2.73) |

Measure of interaction on multiplicative scale: 0.70; 95% CI: [0.54, 0.90]; p = 0.006

Prevalence ratios (PR) are calculated from unadjusted models.

Table S12a. Interaction effects between emphysema and current smoking on prevalence of breathlessness. PR – prevalence ratio

|  | **No emphysema** | | **Emphysema** | |  |
| --- | --- | --- | --- | --- | --- |
|  | N breathlessness /no breathlessness | PR (95% CI) | N breathlessness/no breathlessness | PR (95% CI) | PR (95% CI) for emphysema within smoking strata |
| **Never-smokers** | 459/13,567 | 1 | 8/292 | 0.81 (0.41, 1.62) | 0.81 (0.41, 1.62) |
| **Current smokers** | 180/2,752 | 1.88 (1.59, 2.22) | 76/572 | 3.68 (2.85, 4.51) | 1.71 (1.46, 2.50) |

Measure of interaction on multiplicative scale: 2.34; 95% CI: [1.11, 4.96]; p = 0.026

Prevalence ratios (PR) are calculated from unadjusted models.

Table S12b. Interaction effects between emphysema and current smoking on prevalence of chronic bronchitis. PR – prevalence ratio

|  | **No emphysema** | | **Emphysema** | |  |
| --- | --- | --- | --- | --- | --- |
|  | N chronic bronchitis/no chronic bronchitis | PR (95% CI) | N chronic bronchitis /no chronic bronchitis | PR (95% CI) | PR (95% CI) for emphysema within smoking strata |
| **Never-smokers** | 511/13259 | 1 | 10/286 | 0.92 (0.49, 1.69) | 0.92 (0.49, 1.69) |
| **Current smokers** | 259/2597 | 2.46 (2.14, 2.84) | 94/524 | 4.12 (3.36, 5.06) | 1.68 (0.49, 1.71) |

Measure of interaction on multiplicative scale: 1.83; 95% CI: [0.94, 3.58]; p = 0.07

Prevalence ratios (PR) are calculated from unadjusted models.

Table S12c. Interaction effects between emphysema and current smoking on prevalence of wheeze. PR – prevalence ratio

|  | **No emphysema** | | **Emphysema** | |  |
| --- | --- | --- | --- | --- | --- |
|  | N wheeze/no wheeze | PR (95% CI) | N wheeze/no wheeze | PR (95% CI) | PR (95% CI) for emphysema within smoking strata |
| **Never-smokers** | 650/13,225 | 1 | 15/284 | 1.07 (0.65, 1.76) | 1.07 (0.65, 1.76) |
| **Current smokers** | 504/2,354 | 3.76 (3.38, 4.20) | 151/482 | 5.09 (4.25, 5.96) | 1.35 (1.13, 1.62) |

Measure of interaction on multiplicative scale: 1.26; 95% CI: [0.73, 2.17]; p = 0.39

Prevalence ratios (PR) are calculated from unadjusted models.

Table S13a. Interaction effects between impaired DLco and current smoking on prevalence of breathlessness. PR – prevalence ratio

|  | **No impaired DLco** | | **Impaired DLco** | |  |
| --- | --- | --- | --- | --- | --- |
|  | N breathlessness /no breathlessness | PR (95% CI) | N breathlessness/no breathlessness | PR (95% CI) | PR (95% CI) for impaired DLco within smoking strata |
| **Never-smokers** | 328/12,947 | 1 | 62/689 | 2.77 (2.14, 3.58) | 2.77 (2.14, 3.58) |
| **Current smokers** | 137/2,294 | 1.75 (1.44, 2.11) | 118/755 | 4.53 (3.73, 5.50) | 2.60 (2.03, 3.32) |

Measure of interaction on multiplicative scale: 0.94; 95% CI: [0.65, 1.35]; p = 0.73

Prevalence ratios (PR) are calculated from unadjusted models.

Table S13b. Interaction effects between impaired DLco and current smoking on prevalence of chronic bronchitis. PR – prevalence ratio

|  | **No impaired DLco** | | **Impaired DLco** | |  |
| --- | --- | --- | --- | --- | --- |
|  | N chronic bronchitis/no chronic bronchitis | PR (95% CI) | N chronic bronchitis /no chronic bronchitis | PR (95% CI) | PR (95% CI) for emphysema within smoking strata |
| **Never-smokers** | 489/12,687 | 1 | 31/708 | 1.13 (0.79, 1.61) | 1.13 (0.79, 1.61) |
| **Current smokers** | 209/2349 | 2.20 (1.88, 2.57) | 128/714 | 4.10 (3.42, 4.91) | 3.62 (2.45, 5.26) |

Measure of interaction on multiplicative scale: 1.64; 95% CI: [1.77, 2.52]; p = 0.021

Prevalence ratios (PR) are calculated from unadjusted models.

Table S13c. Interaction effects between impaired DLco and current smoking on prevalence of wheeze. PR – prevalence ratio

|  | **No impaired DLco** | | **Impaired DLco** | |  |
| --- | --- | --- | --- | --- | --- |
|  | N wheeze/no wheeze | PR (95% CI) | N wheeze/no wheeze | PR (95% CI) | PR (95% CI) for emphysema within smoking strata |
| **Never-smokers** | 597/12,605 | 1 | 56/686 | 1.67 (1.28, 2.17) | 1.67 (1.28, 2.17) |
| **Current smokers** | 409/2,168 | 3.51 (3.12, 3.95) | 227/616 | 5.09 (4.25, 5.96) | 1.70 (1.44, 2.00) |

Measure of interaction on multiplicative scale: 1.02; 95% CI: [0.75, 30]; p = 0.92

Prevalence ratios (PR) are calculated from unadjusted models.
